# Divergent Schwann cell–immune cell trajectories across vestibular schwannoma subtypes mirror nerve injury-repair programs

**DOI:** 10.1186/s12974-026-03995-w

**Published:** 2026-08-03

**Authors:** Han-Guang Zhao, Xiao-Ping Wang, Jing Zhang, Si-Kang Ren, Yu-Chao Liang, Hui Li, Jiang-Ming Li, Xiao-Bin Zhao, Lei-Ming Wang, Yuan-Xiang Lin, Zhi Li, Fu Zhao

**Affiliations:** 1 Department of Pediatric Neurosurgery, Beijing Key Laboratory of Drug Innovation for Neuro-Oncology, Beijing Neurosurgical Institute, Capital Medical University, 119 South 4th Ring West Road, Fengtai District, Beijing, 100070 PR China; 2 Department of Neurosurgery, The First Affiliated Hospital of Fujian Medical University, Fuzhou, Fujian province 350005 PR China; 3 Beijing Engineering Research Center of Targeted Drugs and Cell Therapy for CNS Tumors, Beijing, 102600 P.R. China; 4 Department of Neurosurgery, Beijing Tiantan Hospital, Capital Medical University, Beijing, PR China; 5 Department of Nuclear Medicine, Beijing Tiantan Hospital, Capital Medical University, Beijing, PR China; 6 Department of Pathology, Xuanwu Hospital, Capital Medical University, Beijing, PR China; 7 Department of Neurosurgery, Xiamen Hospital of Traditional Chinese Medicine, Xiamen, Fujian Province 361000 China; 8 Department of Neuro-Oncology, Sanbo Brain Hospital, Capital Medical University, Beijing, PR China; 9 Department of Research and Development, Santo Therapeutic co., Xiaoshan District, Hang Zhou, PR China

**Keywords:** Vestibular schwannoma, *NF2*-related schwannomatosis, Molecular subgroups, Macrophage, Tumour microenvironment, Schwann cell

## Abstract

Although Schwann cells (SCs) are increasingly recognised as active contributors to cancer progression, their cell-autonomous oncogenic mechanisms underlying vestibular schwannoma (VS) development remain poorly defined. Moreover, the roles of heterogeneity within the tumour microenvironment (TME) in VS pathogenesis remain unclear. In this study, we performed integrative multi-omic analyses of sporadic VSs and *NF2*-related schwannomatosis (*NF2*-SWN) VSs, using bulk RNA-sequencing, single-nucleus RNA-sequencing (snRNA-seq), DNA methylation profiling, and immunohistochemistry (IHC). Transcriptomic and DNA methylation profiling analyses identified two distinct molecular subgroups, immune-enriched schwannoma (IES) and immune-depleted schwannoma (IDS), independent of syndromic status. We developed multigene and IHC-based classifiers that robustly distinguish the two molecular subgroups. Notably, we found significant differences in clinical characteristics and prognostic outcomes across VS subgroups. Furthermore, snRNA-seq revealed cellular heterogeneity within the VS microenvironment, with phenotypic transitions in SCs and immune cells that closely recapitulated the sequential stages of nerve injury and repair. Collectively, our study reveals that SC plasticity and immune cell dynamics within the tumor microenvironment contribute to intertumoral heterogeneity in vestibular schwannomas, offering insights into the biological processes underlying tumor progression and nerve injury-repair programs.

**Supplementary Information:**

The online version contains supplementary material available at https://doi.org/10.1186/s12974-026-03995-w.

## Introduction

Vestibular schwannomas (VSs) are histologically benign peripheral nerve sheath tumours that originate either spontaneously or as the hallmark of *NF2*-related schwannomatosis (*NF2*-SWN) [1, 2]. Given their anatomical location adjacent to the brainstem and within the cerebellopontine angle (CPA), both tumour progression and surgical intervention carry substantial risks of irreversible neurological morbidity, including hearing loss, facial nerve dysfunction, chronic vestibular imbalance, and in rare cases, mortality [3–5]. To date, no drug therapy has been approved by the US Food and Drug Administration for the treating VSs. Although the vascular endothelial growth factor (VEGF) inhibitor bevacizumab has demonstrated hearing preservation and modest efficacy in reducing tumour volume and slowing growth in a subset of patients with *NF2*-SWN [6], effective treatment strategies remain lacking.

VSs originate from neoplastic Schwann cells (SCs), a type of glial cell in the peripheral nervous system (PNS), harbouring biallelic inactivation of the *NF2* gene and/or chromosome 22q (chr22q loss) [7–9]. Although dysregulation of canonical oncogenic pathways, including the Hippo/Yap, Ras/Raf/MEK/ERK, mTORC1/2, PI3K/AKT, and WNT/β-catenin pathways [10–12], has been extensively characterised upon NF2/Merlin loss, pharmacologic inhibition of these cascades has demonstrated limited clinical efficacy [13–16]. These findings suggest that VSs may exhibit a distinct composition of SC states, potentially accounting for the variability in therapeutic responses and clinical outcomes.

Recent studies employed single-cell and single-nucleus RNA sequencing (snRNA-seq) to characterise the cellular heterogeneity of the tumour microenvironment (TME) in VSs [17–21]. These analyses identified diverse SC subclusters, alongside multiple immune and stromal populations within the VS TME. The cellular composition of the TME enables molecular stratification of VSs into two distinct subgroups: an “immune-enriched” schwannoma (IES) characterised by prominent macrophage infiltration, and an “immune-depleted” schwannoma (IDS) predominantly composed of SCs with minimal immune cellularity [17, 19]. This finding provides critical insights into VS biology and establishes a rationale for integrating molecular classification into the design of future clinical trials. More importantly, given that schwannoma-associated macrophages (SAMs) represent the predominant immune cell population and are implicated in VS progression [22, 23], macrophage-targeted therapeutic approaches, including CSF1/CSF1R inhibitors [17, 18], hold significant translational potential for patients with VS.

Although recent advances have improved our understanding of the cellular architecture of the VS microenvironment, the functional specialisation of SCs and immune cells, including their pathogenic subversion during tumorigenesis, as well as their physiological roles in peripheral nerve injury and repair, remains poorly understood. Moreover, it remains unclear whether sporadic and *NF2*-SWN VSs arise through convergent or divergent developmental mechanisms—a key unresolved question with implications for mechanistic insight and therapeutic stratification. To address these knowledge gaps, we integrated bulk RNA-sequencing, snRNA-seq, and DNA methylation profiling to investigate the phenotypic dynamics and intercellular crosstalk between SCs and immune cells in *NF2*-SWN and sporadic VSs. We identified the extensive intertumoural heterogeneity between *NF2*-SWN and sporadic VSs, highlighting divergent cellular trajectories driving tumourigenesis, even within a shared TME architecture. These insights provide a mechanistic framework to guide the development of molecularly informed therapeutic strategies for VSs.

## Materials and methods

### Ethical approval, consent, and human sample collection

This study was approved by the Ethics Committee of Beijing Tiantan Hospital, Capital Medical University (Nos. KY2020-086-02 and KY2024-128-2) and The First Affiliated Hospital of Fujian Medical University (No. FMU [2024]588). Patients with a histopathological diagnosis of schwannoma and available clinical data were included in this study. All patients underwent preoperative contrast-enhanced brain magnetic resonance imaging (MRI) for the diagnosis of VSs. All samples were collected during surgery after obtaining informed consent. Samples were stored as fresh-frozen or formalin-fixed paraffin-embedded (FFPE) tissue. Individuals were diagnosed with *NF2*-SWN according to the updated diagnostic criteria [24]. Hearing function was classified according to the American Academy of Otolaryngology–Head and Neck Surgery (AAO-HNS) criteria. Facial nerve function was assessed using the House–Brackmann grading scale.

### Bulk RNA-sequencing

Bulk RNA-sequencing was conducted on VS specimens. Total RNA was extracted from specimens using the RNeasy Mini Kit (Qiagen, Germany, catalog #74004) according to the manufacturer’s instruction. RNA integrity was assessed using the Agilent Bioanalyzer 2100 system (Agilent Technologies, USA); only samples with RNA integrity number (RIN) ≥ 7.0 were included for downstream library construction. cDNA libraries were prepared using the TruSeq RNA Sample Prep Kit (Illumina, USA) following adapter ligation and reverse transcription PCR amplification. Paired-end 150-bp reads were generated on the Illumina NovaSeq 6000 platform (Illumina, USA). Raw FASTQ files were subject to quality control (QC) using FastQC (v0.11.9). Low-quality bases and adapter sequences were trimmed using Trimmomatic (v0.39) with the following parameters: *ILLUMINACLIP: TruSeq3-PE.fa:2:30:10 LEADING:3 TRAILING:3 SLIDINGWINDOW:4:15 MINLEN:36*. Post-trimming QC confirmed that all samples achieved > 90% of bases with Phred quality score ≥ Q30. The mean raw sequencing depth was 42.5 ± 4.8 million reads per sample (range: 34.8–50.1 million). The mean Q20 base quality ratio was 94.3 ± 0.2% (range: 93.7–94.7%). After adapter trimming and quality filtering, the mean number of clean reads was 39.5 ± 3.9 million per sample (mean clean ratio: 93.0 ± 0.5%; range: 92.1–93.8%). Ribosomal RNA (rRNA) contamination was extremely low (mean rRNA ratio: 0.15 ± 0.11%; range: 0.05–0.51%), yielding a mean of 39.4 ± 3.9 million rRNA-depleted reads per sample (range: 32.3–46.7 million).

Trimmed reads were aligned to the human reference genome GRCh38.p14 using HISAT2 (v2.0.4) with default parameters for spliced alignment. HISAT2’s splice-aware mapping algorithm allows split reads that cannot be fully matched to be segmented and mapped, which is particularly suitable for eukaryotic transcriptome sequencing data with intron-exon junctions. The alignment output was generated in BAM format. The mean overall mapping rate was 97.3 ± 0.4% (range: 96.7–97.9%). The mean unique mapping rate was 97.1 ± 0.4% (range: 96.5–97.8%), with multi-mapped reads accounting for only 2.9 ± 0.4% of total aligned reads. The mean number of uniquely mapped reads was 37.5 ± 3.7 million per sample (range: 30.2–43.7 million). Properly paired reads constituted the majority of alignments (mean paired mapping rate: 96.8 ± 0.5%).A total of 63,241 genes were annotated in the reference; after filtering for genes with ≥ 1 count per million (CPM) in at least 2 samples, 18,642 genes were retained for downstream analysis. The mean number of detected genes per sample was 15,823 ± 1,247 (range: 13,102–18,104). Raw count data were imported into R (v4.2.1) using the edgeR package (v3.36.0). Lowly expressed genes were filtered using the criterion of CPM > 1 in at least 2 samples. Library size normalization was performed using the trimmed mean of M-values (TMM) method implemented in edgeR. Normalized counts were converted to counts per million (CPM) and log2-transformed with a pseudo-count of 1 (log2[CPM + 1]).

ConsensusClusterPlus (v1.48.0) was applied to the top 1,000–4,000 most variable genes (ranked by median absolute deviation of log2-CPM values) to determine the optimal cluster number (k). The algorithm was executed with 1,000 resampling iterations, 80% gene and sample subsampling fractions, and Pearson correlation as the distance metric. The cophenetic correlation coefficient and delta area under the cumulative distribution function curve were used to select the optimal k. Silhouette width analysis further validated cluster stability. Non-negative matrix factorization (NMF, R package NMF v0.23.0) was performed on the same variable gene sets across 2,000 permutation iterations for cross-method validation. Concordance between ConsensusClusterPlus and NMF assignments was quantified using the adjusted Rand index.

Inter-group differential expression analysis was conducted using DESeq2 (v1.30.1) on raw count data prior to ComBat-based batch effect correction. Sequencing batch was explicitly modeled as a covariate in the design formula (~ batch + phenotype) to account for technical variability. Genes exhibiting an adjusted p-value (false discovery rate, FDR) < 0.05 and |log₂(fold change)| > 1 were deemed statistically significant. Subsequently, Gene Set Enrichment Analysis (GSEA) was performed with the clusterProfiler R package (v3.18.1) to identify biologically coherent pathways and functional categories enriched among differentially expressed genes. Unlike over-representation analysis—which tests for enrichment only among pre-selected significant genes—GSEA evaluates the distribution of gene set members across the entire ranked list of genes (ordered by log₂ fold change from DESeq2), thereby increasing sensitivity to coordinated but modest expression shifts. All protein-coding genes were ranked in descending order based on their DESeq2-derived log₂ fold change values. Enrichment testing was carried out against two authoritative annotation resources: (1) Gene Ontology (GO): Biological Process (BP), Molecular Function (MF), and Cellular Component (CC) terms were assessed using the gseGO function with the org.Hs.eg.db annotation package (v3.12.0), sourced from Bioconductor release 3.14; (2) Kyoto Encyclopedia of Genes and Genomes (KEGG): Pathway-level enrichment was evaluated using the gseKEGG function, interfacing with the KEGG database (2021 release) via the KEGGREST API, specifying organism = “hsa” (Homo sapiens). For both GO and KEGG analyses, standardized parameters were applied: 1,000 permutations (nPerm) for empirical p-value estimation; Benjamini–Hochberg (BH) adjustment (pAdjustMethod = “BH”) for multiple testing correction; significance thresholds of *P*-value < 0.05 and q-value < 0.2; and statistical significance defined as adjusted *P*-value < 0.05 combined with |normalized enrichment score (NES)| > 1. A positive NES indicates relative upregulation of the pathway in the test group compared to the reference group; a negative NES indicates relative downregulation. Visualization of enrichment results included GSEA enrichment plots (generated via gseaplot2) and functional category dot plots (dotplot), both produced using clusterProfiler.

### DNA methylation profiling analysis

DNA methylation data were generated and pre-processed as we described previously [17]. Briefly, genomic DNA from VS samples was profiled on the Illumina Infinium MethylationEPIC BeadChip (850 K). Raw IDAT files were processed in R (v4.3.0) using the minfi package (v1.46.0), with Noob normalisation for background correction and dye-bias adjustment. Methylation levels were quantified as β-values [M / (M + U + 100)], ranging from 0 (unmethylated) to 1 (fully methylated). Quality-control filtering was applied at both sample and probe levels. Samples were excluded if the mean detection *P*-value exceeded 0.01 or if > 1% of probes failed (detection *P*-value > 0.01 and bead count < 3). Sex was inferred from X- and Y-chromosome probe intensities (minfi getSex) and cross-checked against clinical records; no discordant samples were detected. Genotype concordance across matched multi-omics aliquots (methylation array, bulk RNA-seq, and scRNA-seq from the same patient) was verified using the 59 SNP probes embedded on the EPIC array; perfect concordance confirmed sample identity and excluded cross-contamination or swaps. Probes mapping to sex chromosomes, overlapping known SNPs (MAF > 1%), or classified as cross-reactive were removed. Probes with missing β-values in > 20% of samples were excluded; sporadic missing values in the retained data were imputed by k-nearest neighbour (k = 10) prior to downstream analysis.

Differential methylation, clustering, and functional enrichment. To harmonise with RNA-seq annotations (GRCh38), CpG coordinates were converted from hg19 to hg38 using UCSC liftOver (chain file: hg19ToHg38.over. chain). Differentially methylated positions (DMPs) were identified using limma (|Δβ| > 0.15, FDR < 0.05) and annotated to genomic features (promoters, gene bodies, CpG islands) using the hg38-lifted manifest. Unsupervised clustering was performed on the 1,000 most variable CpG sites, visualised by heatmap with per-row Z-score scaling. Classification robustness was evaluated by non-negative matrix factorisation (NMF; R package v0.23.0) on the 5,000 most variable CpG sites across 1,000 permutation iterations; concordance between methods was quantified using the adjusted Rand index. t-SNE dimensionality reduction (Rtsne v0.15) was performed independently. External validation used a publicly available VS dataset (GSE56596) from the Gene Expression Omnibus (GEO). Gene set enrichment analysis (GSEA) was performed with clusterProfiler against MSigDB Hallmark and KEGG collections, ranking genes by differential methylation significance (–log10[P] × sign[Δβ]). Pathways with normalised enrichment score (NES) > 1.0 and FDR < 0.25 were deemed significant.

### Establishment of multigene classifier

NMF consensus clustering on TMM-normalized, log2-transformed CPM values were performed on the training cohort to identify two robust molecular subgroups of VS (IES and IDS). The Boruta algorithm (random-forest-based feature selection) was applied to identify the top 30 optimal signature genes that maximally discriminated between the two subgroups. For classifier development, the random forest algorithm (R package ‘randomForest’, v4.6-14) was used to extract discriminative gene signatures from the training cohort. The model comprised 500 decision trees, with features ranked by mean decrease in Gini index. The optimal 30-gene classifier was defined through iterative feature selection maximizing the out-of-bag error rate. To identify a minimal gene set, we performed forward selection starting from the 30-gene signature. Beginning with the single gene yielding the highest classification accuracy, we iteratively added the next most informative gene and evaluated performance using 10-fold cross-validation. The optimal reduced classifier was defined as the minimal gene set capable of discriminating between the two molecular subgroups with accuracy comparable to that of the full 30-gene signature; it was derived using forward selection (R package caret, version 6.0–86).

Comprehensive validation was performed across independent datasets. Internal robustness was assessed by 10-fold cross-validation within the training cohort. For external validation, we employed three publicly available VS transcriptomic datasets (GSE141801, GSE54934, and GSE108524) from the Gene Expression Omnibus, together with the *NF2*-SWN VS transcriptomic dataset (GSA-Human: HRA002762). To mitigate platform-specific biases, all validation datasets were harmonized through quantile normalization, log2 transformation, and ComBat batch correction (sva package, v3.38.0) prior to classifier application. We employed a centroid-based nearest-template prediction strategy for label transfer. The median expression centroid (30-gene vector) was computed for molecular classification in the training set. For each validation sample, the Spearman correlation coefficient was computed between its 30-gene expression profile and the centroid vectors of the two subgroups. Each sample was assigned to the subgroup corresponding to the higher Spearman correlation coefficient. All expression data were log2-transformed prior to analysis: log2(x + 1) for raw FPKM/TPM values.

Silhouette analysis was performed across k = 2–8 clusters using NMF (R package NMF, v0.21.0), K-means, PAM-Cor, and HC-Ward (R package cluster, v2.1.0); the optimal cluster number was determined by the highest mean silhouette score (R package factoextra, v1.0.7). Performance was quantified using accuracy, sensitivity, specificity, positive and negative predictive values, and the area under the receiver operating characteristic curve (AUC-ROC) via the ‘caret’ (v6.0-86) and ‘pROC’ (v1.17.0) packages in R (v4.1.0).

### Droplet-based snRNA-seq analysis

High-throughput snRNA-seq was performed on frozen VS samples, as previously described [17]. In brief, nuclear suspensions were prepared according to the manufacturer’s protocol using the Nucleus Isolation Kit (Shbio, catalogue #52009-10, Shanghai, China). Droplet encapsulation was performed on the 10x Genomics Chromium controller using the Single Cell 3’ v3.1 Library and Gel Bead kit. Libraries were sequenced in paired-end 150-bp mode on the Illumina NovaSeq 6000 platform (Illumina, CA, USA), with a median coverage of 30,000 reads per nucleus. The QC metrics for each sample are summarized in Supplementary data file 2. The median number of unique molecular identifiers (UMIs) per nucleus ranged from 1,990 to 3,470 (mean ± SD: 3,354 ± 573), and the median number of detected genes per nucleus ranged from 1,111 to 1,805 (mean ± SD: 1,673 ± 268). The median mitochondrial gene percentage ranged from 0.067% to 0.535% (mean ± SD: 0.26% ± 0.16%). Cells were filtered based on the following criteria: (i) UMI count between 500 and 10,000; (ii) gene count between 400 and 5,000; and (iii) mitochondrial gene percentage < 10%. Gene expression matrices were normalized using the LogNormalize method with a scale factor of 10,000. Highly variable genes were identified using the FindVariableFeatures function (selection method: “vst”, nfeatures = 2,000). Batch correction was performed using CCA to account for inter-sample variability. Principal component analysis (PCA) was conducted on the integrated data, and the top 30 principal components were used for Uniform Manifold Approximation and Projection (UMAP) visualization and clustering. Unsupervised clustering was performed using the Seurat FindNeighbors and FindClusters functions (resolution = 0.5). Cluster-specific marker genes were identified using the Wilcoxon rank-sum test (FindAllMarkers, min.pct = 0.25, logfc.threshold = 0.25). Cell types were annotated based on the expression of established lineage-specific markers and cross-referenced with published scRNA-seq datasets (GSE216783, HRA002762). Label transfer was performed using Seurat’s FindTransferAnchors and TransferData algorithms.

Differentiation trajectories were reconstructed using Monocle 2 (v2.22.0). Dimensionality reduction was performed with DDRTree. Root states were defined based on prior biological knowledge of Schwann cell differentiation hierarchy and validated by correlation with known lineage markers. Pseudotime values were computed using order_cells, and branch-dependent genes were identified via differentialGeneTest (fullModelFormulaStr = “~sm.ns(Pseudotime)”). Trajectories were visualized using plot_cell_trajectory with coloration indicating cellular state and pseudotime. Slingshot (v2.0.0) was applied for principal curve-based cyclic pseudotime inference and validated the trajectory topology using a PAGA-like graph-based approach.

### Immunohistochemistry

Immunohistochemistry (IHC) staining was performed on FFPE samples using a Leica Bond RXm automated staining processor (Leica Biosystems, Buffalo Grove, IL), as previously described [25]. Briefly, tissue blocks were cut into 4-µm sections, fixed on slides, and dried at 37 °C for 12–24 h. Sections were incubated with primary antibodies against ionised calcium-binding adaptor molecule-1 (Iba1) (1:2000; CST, catalogue #17198, Massachusetts, US), CD68 (1:400; CST, catalogue #76437), and CD163 (1:400; CST, catalogue #93498), at 4 ℃ overnight. After incubation with a secondary antibody (ZSGB-BIO, catalogue #PV-6000, Beijing, China) for 30 min at 37 °C, the signal was visualised using a DAB Chromogenic Kit (Dako). TIFF images of the IHC staining were captured from each core at 40× magnification and quantified using the Aperio ScanScope XT slide scanner (Leica Biosystems, Wetzlar, Germany) slide scanner, with slides digitised at 20× magnification.

### Digital quantification of hematoxylin-eosin and IHC staining

Quantification of Antoni A and Antoni B areas was performed using ImageJ (National Institutes of Health, Bethesda, MD, USA). Whole-slide hematoxylin-eosin (HE) staining images were imported into ImageJ, and the total tissue area was measured by outlining the entire section boundary. Within each section, Antoni A (hypercellular, spindle-cell-rich) and Antoni B (hypocellular, loosely arranged) regions were manually delineated separately according to established histomorphological criteria, and the area of each region was quantified. The Antoni A-to-Antoni B area ratio was subsequently calculated for each sample.

A multiplatform analytical workflow was established to assess immunopositivity for Iba1, CD68, and CD163. Whole-slide images were acquired at 20× magnification using a digital slide scanner (Aperio AT2, Leica Biosystems). QuPath-based analysis (v0.4.3) was performed for automated cell detection and classification [26]. Initial cell detection parameters were optimised using 5–10 representative slides with manual pathologist review. StarDist, a deep learning-based nuclei segmentation algorithm [27], was integrated into the QuPath workflow to enable precise cellular boundary identification. Cells were classified into four intensity categories based on DAB optical density (OD): negative (OD < 0.2), weak (0.2 ≤ OD < 0.4), moderate (0.4 ≤ OD < 0.6), and strong (OD ≥ 0.6). Positive cells were defined as those with weak, moderate, or strong staining (OD ≥ 0.2). The percentage of positive cells was calculated as (positive cells/total detected cells) × 100%.

### Immunofluorescence (IF) staining

FFPE sections were deparaffinised in xylene and rehydrated through a series of graded ethanol washes. The tissue was subjected to antigen retrieval using Tris-EDTA buffer (pH 9), followed by quenching with hydrogen peroxide and blocking with 5% goat or donkey serum. The following primary antibodies were employed: S100β (1:300; Proteintech, Wuhan, China, catalogue #15146-1-AP), matrix metalloproteinase 9 (MMP9,1:300; catalogue #10375-2-AP, Proteintech), CD8 (1:1000; Proteintech catalogue #66868-1-Ig), and PD1 (1:1000; Proteintech, catalogue #18106-1-AP). After being washed with PBS, the sections were incubated with Alexa Fluor 594-conjugated goat anti-rabbit IgG (1:300; ZSGB-bio, catalogue #ZF-0516) or Alexa Fluor 488-conjugated goat anti-mouse IgG (1:300; ZSGB-bio, catalogue #ZF-0512) for 1 h. Nuclei were visualised using DAPI staining. A Zeiss AxioScan.Z1 slide scanner (bright field, 40× magnification, 0.25 μm/pixel resolution) was used to scan the whole digital slides, and the data were quantified using the Aperio ScanScope XT platform. For the MMP9 and PD1 staining analyses, the entire slide was captured as 16-bit tiled TIFF images. Colour deconvolution was applied to each image to isolate the region of interest (ROI); non-tumour areas and artefacts were excluded. The proportion of co-expressing positive cells was calculated based on the statistical data of the ROIs.

### Establishment of an IHC-based classifier

A logistic regression classifier was developed for molecular subgroup prediction. Model construction employed the ‘glm’ function in R (v4.1.0) with binomial family specification, using molecular subgroup designation (IES vs. IDS) as the binary outcome. Discriminative performance was assessed via ROC curve analysis using the ‘pROC’ package (v1.17.0), with the AUC and 95% confidence intervals (Cis) derived using bootstrap resampling (*n* = 2000 iterations). The optimal classification threshold was determined by maximising the Youden index (sensitivity + 1 − specificity). Validation was performed on an independent cohort of VSs processed using identical immunohistochemical and quantification protocols. Model accuracy in the validation cohort was evaluated using classification metrics, including sensitivity, specificity, positive predictive value, negative predictive value, and overall accuracy. Calibration was verified through comparison of predicted probabilities with observed outcomes using calibration plots and Brier score calculations.

### Statistical analysis

Binary and categorical variables were compared using the two-tailed Chi-square (χ²) test or Fisher’s exact test. Continuous variables are presented as mean ± SD and compared using the two-tailed Student’s t-test. One-way analysis of variance (ANOVA) was employed for comparisons among three or more independent groups when residuals were normally distributed; otherwise, the nonparametric Kruskal–Wallis test was applied. The false discovery rate (FDR) was controlled using the Benjamini–Hochberg procedure. Spearman’s correlation (non-parametric) with FDR correction was applied for correlation analysis. All statistical analyses were performed using GraphPad Prism V9.0 (GraphPad Software, San Diego, CA, USA). Statistical significance was set at *P* < 0.05.

## Results

### Patient cohort

**Fig. 1:**
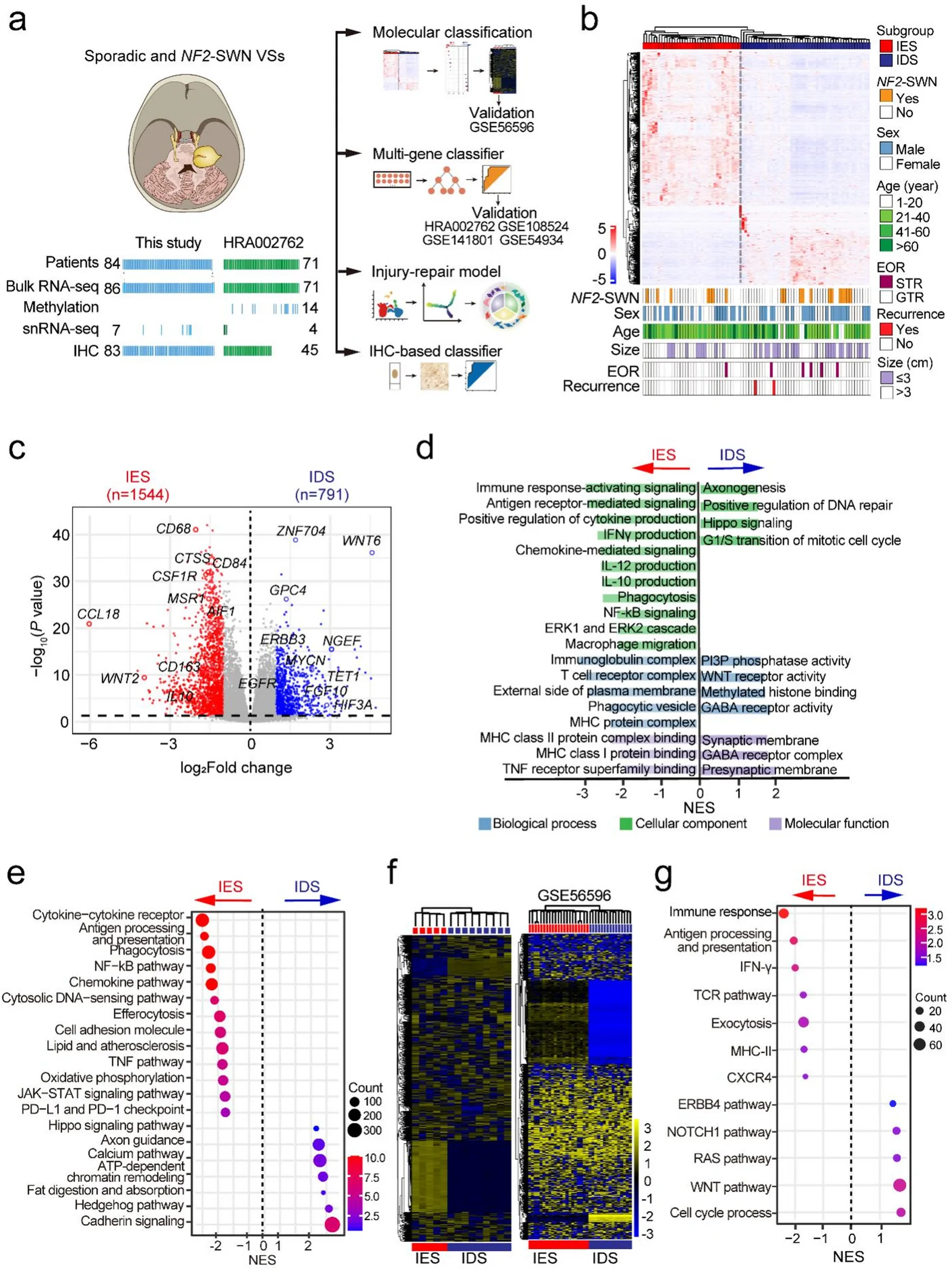
Integrative transcriptomic analysis delineates two molecular subgroups in vestibular schwannomas (VSs). **a** Workflow schematic diagram depicting multi-platform profiling of sporadic and *NF2*-SWN VSs for bulk RNA-sequencing, DNA methylation profiling, single nucleus RNA sequencing, and immunohistochemistry (IHC). **b** Hierarchical clustering of 1,500 high-variance transcripts partitions samples into two principal clusters. Right-sided annotations display clinical covariates. Abbreviations: IES, immune-enriched vestibular schwannoma; IDS, immune-depleted vestibular schwannoma; GTR, gross total resection; STR, subtotal resection. **(c)** Volcano plot of differentially expressed genes (DEGs) between two molecular subgroups of VSs. The x-axis represents log₂ fold-change; the y-axis represents –log_10_(*P* value). DEGs (|log_2_FC| > 1.2, FDR-adjusted *P* < 0.05) are highlighted; the top significant genes are labelled. *P* values were calculated using the two-sided Wilcoxon rank-sum test. **d **Gene Set Enrichment Analysis (GSEA) of GO terms between IES and IDS. NES, normalized enrichment score. Blue, biological process; green, cellular component; purple, molecular function. **e** GSEA of KEGG pathways between IES and IDS. Normalised enrichment scores (NES) are shown; significance was determined using the Kolmogorov–Smirnov test with FDR correction. **f** DNA methylation-based hierarchical clustering of 1,000 variable CpG loci in 14 *NF2*-SWN VSs and external dataset (GSE56596). **g** GSEA of differentially methylated genes between IES and IDS subgroups using the GO database. Normalised enrichment scores (NES) are shown; significance was determined using the Kolmogorov–Smirnov test with FDR correction

This study enrolled 86 VSs specimens obtained from 84 patients (67 patients with sporadic VSs and 17 patients with *NF2*-SWN); of these, two additional specimens were collected from distinct intratumoral regions in two separate patients (Fig. 1a; Supplementary Table 1; Supplementary Material 1). The male-to-female ratio was 0.95:1. The mean age at the time of surgery was 51.8 years for patients with sporadic VSs, which was significantly higher than the 30.2 years observed in those with *NF2*-SWN (*P* < 0.001; Supplementary Fig. 1a). The mean tumour size was similar between sporadic VSs and *NF2*-SWN VSs (32.2 mm [range: 12.0–63.1] vs. 35.1 mm [range: 15.0–55.8]).

### The transcriptional landscape identifies two subgroups in VSs regardless of syndromic status

We performed bulk RNA-sequencing on all VS samples to compare the gene expression profiles between sporadic and *NF2*-SWN VSs (Fig. 1a). Unsupervised hierarchical clustering (UHC) analysis revealed two robust clusters in VSs, irrespective of syndromic status (Fig. 1b). This indicates a shared tumourigenic and developmental trajectory between sporadic and *NF2*-SWN VSs. NMF, consensus clustering matrix, and t-SNE analyses collectively corroborated the presence of two distinct molecular clusters (Supplementary Fig. 1b–d). We found that samples obtained from distinct anatomical regions of the same VS exhibited high transcriptional similarity and consistently clustered together (Supplementary Fig. 1d), suggesting low intratumoural heterogeneity in VSs. No significant difference was observed in the Antoni A-to-Antoni B area ratio between the two molecular subgroups, indicating that the molecular subgroups capture heterogeneity beyond classical histopathological patterns and are driven by distinct transcriptomic programs rather than structural architecture (Supplementary Fig. 1e, f).

To identify biological processes and signalling pathways distinguishing two molecular subgroups of VSs, we performed differential gene expression and GSEA using transcriptome profiles (Fig. 1c–e). GO and KEGG enrichment analyses revealed divergent activation patterns across canonical signaling pathways: one subgroup showed significant upregulation of immune-related pathways, while the other exhibited pronounced enrichment in cell cycle, proliferation, and metabolic pathways. Based on their distinct, pathway-enriched biological activities, the two subgroups were continued designated as IES and IDS, as we discribed previously.

To investigate whether transcriptome-defined molecular subgroups are associated with distinct epigenetic signatures, we performed an integrative analysis of the DNA methylation and matched transcriptomic data. UHC, NMF, and t-SNE analyses of our cohort (*n* = 13) and the publicly available dataset (GSE56596) revealed perfect segregation of transcriptome-defined molecular subgroups, with each subgroup forming a distinct, cohesive methylation cluster (Fig. 1f; Supplementary Fig. 1g–i). This indicates that the two subgroups of VSs harbour not only divergent transcriptional programmes but also distinct epigenetic profiles. Moreover, Functional analysis of DNA methylation profiles further validated the immune-enriched signature of IESs and the proliferative signature of IDSs (Fig. 1g; Supplementary Fig. 1j), suggesting that differential DNA methylation patterns potentially serve as foundational regulatory mechanisms establishing the distinct biological identities of IESs and IDSs.

### Development of a multigene classifier for molecular subgrouping

**Fig. 2:**
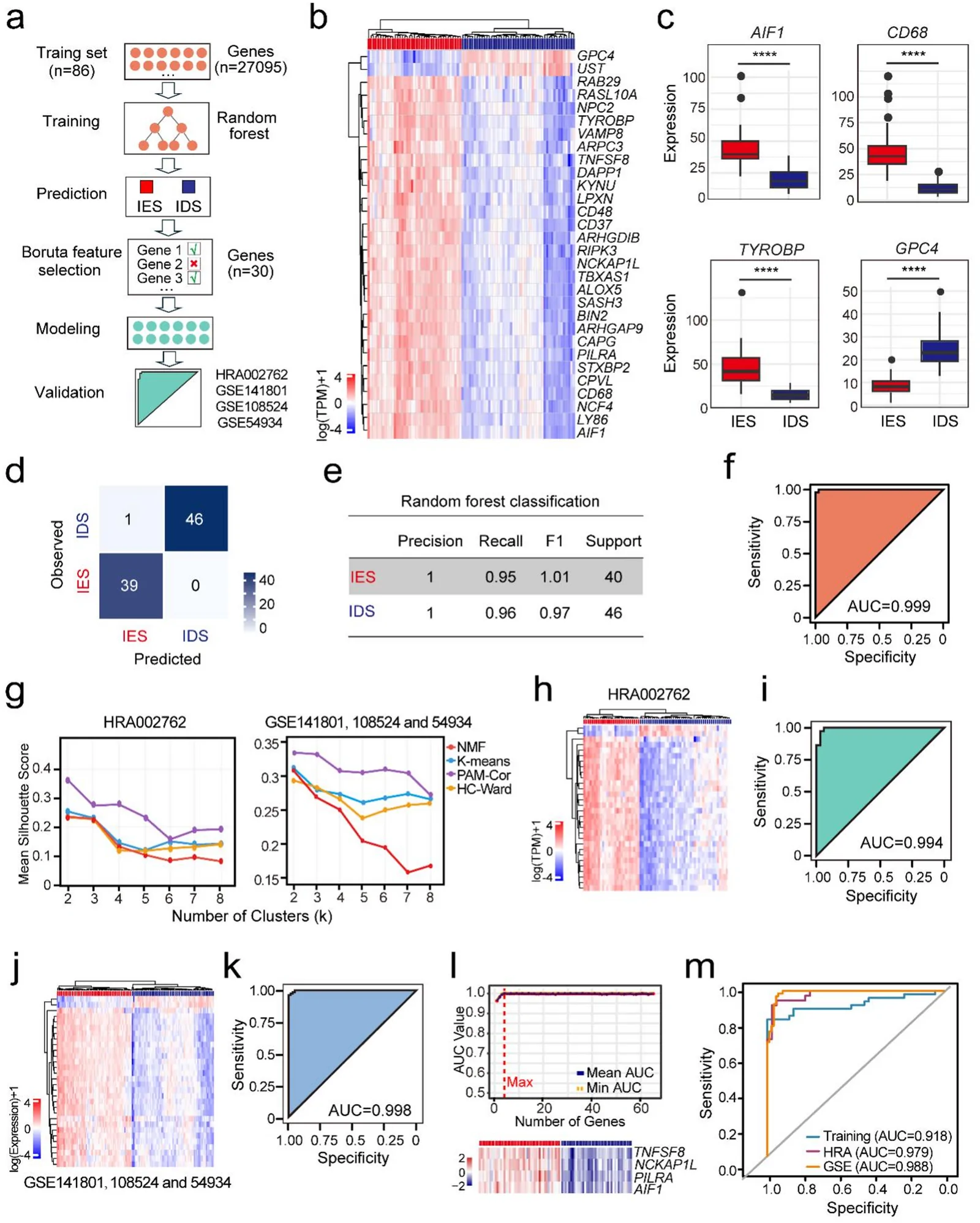
A 30-gene based classifier for molecular subgroup diagnosis in sporadic and *NF2*-SWN VSs. **a** Study design and analytical workflow. The classifier was trained on 86 samples (*n* = 27,095 genes) using random forest, followed by Boruta feature selection (*n* = 30 genes) and validation across four external cohorts (HRA002762, GSE141801, GSE108524, and GSE54934). **b** Heatmap of the 30 classifier genes across the training cohort. **c** Differential expression of representative classifier genes (*AIF1*, *CD68*, *TYROBP*, and *GPC4*) between IES and IDS subgroups. Expression values are log_2_(TPM + 1). Box plots display median (horizontal line) and interquartile range (IQR); whiskers extend to 1.5× IQR. Statistical significance: ****P < 0.0001 (two-sided Wilcoxon rank-sum test). **d** Confusion matrix from leave-one-out cross-validation in the training cohort. Rows: observed labels; columns: predicted labels. **e** Random forest classification metrics for IES and IDS subgroups. Precision, recall, F1-score, and sample support (n) are shown. **f** Receiver operating characteristic (ROC) curve of the 30-gene classifier in the training cohort. **g** Silhouette analysis across k = 2–8 clusters using NMF, *K*-means, PAM-Cor, and HC-Ward in HRA002762 (left) and combined external datasets (right). **h** Heatmap analysis 30 classifier genes in our previously published *NF2*-SWN RNA-seq dataset (HRA002762). **i** ROC curve in HRA002762. **j** Heatmap of the 30 classifier genes across three independent external datasets (GSE141801, GSE108524, and GSE54934). **k** ROC curve of the classifier in the combined external validation cohort. **l** Forward selection to identify minimal gene set. Top, AUC as a function of gene number; bottom, heatmap of top 4 genes (*TNFSF8*, *NCKAP1L*, *PILRA*, and *AIF1*). **m** ROC curves of training (AUC = 0.918), HRA002762 (AUC = 0.979), and combined GSE cohorts (AUC = 0.988)

To identify a set of genes that could enable the prospective classification of VSs into the two molecular subgroups, we applied the random forest algorithm to screen for the most discriminative subgroup-specific genes in the training cohort (Fig. 2a). The top 30 most DEGs, such as *AIF1*, *CD68*, and *TYROBP* for IESs, and *GPC4* for IDSs, were integrated into a multigene classifier model (Fig. 2b, c; Supplementary Fig. 2). The confusion matrix demonstrated high concordance between classifier predictions and the ground-truth labels (Fig. 2d, e). ROC analysis demonstrated excellent discriminatory performance in the bootstrap internal validation, with an AUC of 0.999 (Fig. 2f).

We subsequently employed four independent external validation cohorts of VSs (HRA002762, GSE141801, GSE108524, and GSE54934) to assess model generalizability. Silhouette analysis indicated that a two-cluster solution provided the optimal partitioning of samples across these datasets (Fig. 2g). ROC analysis confirmed the robust performance of our classifier in molecular subgrouping across these datasets (Fig. 2h–k). Through forward selection, we further identified a four-gene classifier, comprising *TNFSF8*, *NCKAP1L*, *PLRA*, and *AIF1*, that achieves discriminative performance comparable to that of the 30-gene signature (Fig. 2l, m). Collectively, these results demonstrate that the multigene classifier reliably discriminates between the molecular subgroups of VS.

### Identification of clinically relevant differences among the four VS subtypes

We identified significant differences in clinical characteristics across the four VS subtypes (*NF2*-SWN IES, sporadic IES, *NF2*-SWN IDS, and sporadic IDS) (Table 1; Supplementary data file 1). Patients with *NF2*-SWN IDS were significantly younger at the time of surgery and exhibited a higher prevalence of preoperative facial paralysis compared with those with the other subtypes (both *P* < 0.001). By contrast, patients with sporadic IDSs had the smallest tumour size at surgery (*P* = 0.045). Gross total resection (GTR) was achieved on all sporadic IESs with excellent preservation of facial nerve function (House–Brackmann grades Ⅰ–Ⅲ). By comparison, GTR was achieved in only 75.5% of *NF2*-SWN IDSs (*P* = 0.004), and 49.0% of this group patients exhibited severe postoperative facial weakness (House–Brackmann grades Ⅵ–Ⅳ) (*P* < 0.001). Furthermore, patients with *NF2*-SWN IDSs exhibited the highest tumor recurrence rate (18.4%; *P* = 0.007). These findings demonstrate pronounced clinical heterogeneity among the four VS subtypes, highlighting the critical need to further elucidate subtype-specific biological mechanisms to refine risk stratification and guide personalised therapeutic strategies.

**Table 1:** Comparative analysis of clinical characteristics across the four VS subtypes

Characteristic	*NF2*-SWN IES (*n* = 40)	Sporadic IES (*n* = 33)	*NF2*-SWN IDS (*n* = 49)	Sporadic IDS (*n* = 33)	*P* value*
Tumor size (mm)					0.045
≤ 30	9 (29.0%)	9 (27.3%)	19 (38.8%)	17 (51.5%)	
> 30	31 (71.0%)	24 (72.7%)	30 (61.2%)	16 (48.5%)	
Age at surgery					< 0.001
1‒20 yrs	5 (12.5%)	0 (0%)	18 (36.7%)	0 (0%)	
20‒40 yrs	29 (72.5%)	5 (15.2%)	24 (49.0%)	3 (9.1%)	
> 40 yrs	6 (15.0%)	28 (81.8%)	7 (14.3%)	30 (90.9%)	
Pre. hearing function^†^					0.442
Class A	1 (2.5%)	1 (3.0%)	0 (0%)	2 (6.1%)	
Class B	10 (25.0%)	8 (24.2%)	10 (20.4%)	4 (12.1%)	
Class C	8 (20.0%)	9 (27.3%)	11 (22.4%)	4 (12.1%)	
Class D	21 (52.5%)	15 (45.5%)	28 (57.2%)	23 (69.7%)	
Pre. facial paralysis					< 0.001
Yes	1 (2.5%)	1 (3.0%)	15 (31.2%)	2 (6.1%)	
No	39 (97.5%)	32 (97.0%)	33 (68.8%)	31 (93.9%)	
Extent of resection					0.004
GTR	37 (92.5%)	33 (100%)	37 (75.5%)	29 (87.8%)	
STR	3 (7.5%)	0 (0%)	12 (24.5%)	4 (12.2%)	
Post. hearing function^†^					0.097
Class A	0 (0%)	0 (0%)	1(2.0%)	0 (0%)	
Class B	1 (2.5%)	1 (3.0%)	0 (0%)	3 (9.1%)	
Class C	3 (7.5%)	8 (24.2%)	4 (8.2%)	4 (12.1%)	
Class D	36 (90.0%)	24 (72.7%)	44 (89.8%)	26 (78.8%)	
Post. facial weakness					< 0.001
1–3	30 (75.0%)	33 (100%)	25 (51.0%)	23 (69.7%)	
4–6	10 (25.0%)	0 (0%)	24 (49.0%)	10 (30.3%)	
Recurrence					
No	39 (97.5%)	33 (100%)	40 (81.6%)	31 (93.9%)	0.007
Yes	1 (2.5%)	0 (0%)	9 (18.4%)	2 (6.1%)	

*DOS* duration of symptoms, *GTR* gloss total removal, *Pre.* preoperative, *Post.* postoperative, *STR* subtotal resection, *yrs* years

^*^ *P* value was calculated by Chi-square (χ2) or Fisher’s exact test

^†^ Hearing function was assessed using the AAO-HNS hearing classification

A cohort of 70 patients with *NF2*-SWN from our previously published study was included for statistical analysis [17]

### snRNA-seq analysis reveals cellular heterogeneity across four VS subtypes

**Fig. 3:**
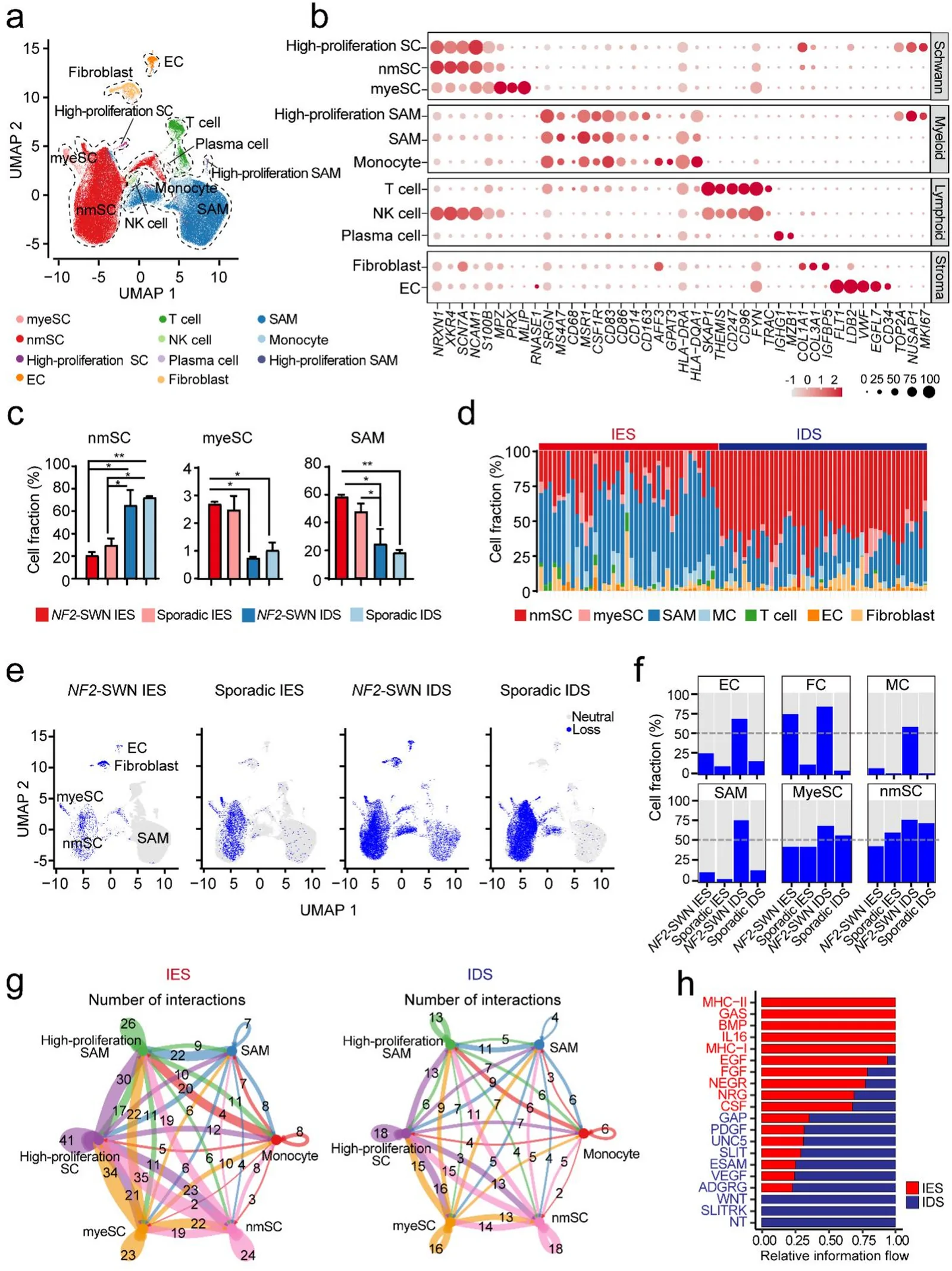
Single-nucleus RNA sequencing (snRNA-seq) reveals distinct cellular compositions across four molecular subtypes of VSs. **a** Uniform manifold approximation and projection (UMAP) visualisation of 131,829 high-quality single nuclei from 11 VS samples. Cell types: *myeSC* myelinating SC, *nmSC* non-myelinating SC, *SAM* schwannomas-associated macrophage, *MC* monocyte, *NK* natural killer cell, *EC* endothelial cell. **b** Dot plot showing expression levels of selected marker genes (x-axis) across cell types (y-axis). Colour intensity indicates average expression; dot size represents the percentage of expressing cells. **c **Cell type proportions across four molecular subtypes of VSs. Data are presented as mean ± SEM. Statistical significance: *P < 0.05, **P < 0.01 (two-way ANOVA with Tukey’s post-hoc test). **d** Stacked bar plot depicting cell type composition in IESs versus IDSs, estimated by CIBERSORTx deconvolution of bulk RNA-sequencing data. **e** UMAP plots highlighting cells with inferred chr22q loss (blue) in four molecular subtypes. Grey indicates neutral copy number. **f** Proportion of chr22q loss across cell types in each molecular subtype. **g** Circle plots showing the strength among different cell types in IES (left) and IDS (right). Circle sizes are proportional to the number of cells in each cell group and edge width represents the communication probability. **h** Relative information flow of top 10 signaling pathways in IES (red) and IDS (blue). Information flows, sum of communication probability across all cell pairs in each network

To characterise cellular heterogeneity within the TME across the four VS subtypes, we performed snRNA-seq analysis on three sporadic IESs, three sporadic IDS, and one *NF2*-SWN IDS. We retained 94,126 high-quality cells for downstream transcriptional analysis, with an average of 13,446 cells per sample (Supplementary Fig. 3a, b; Supplementary Material 2). A total of 37,703 high-quality cells derived from four *NF2*-SWN VSs (two IDS and two IES; Genome Sequence Archive: HRA002762) were also included in the analysis [17]. A comprehensive integration of high-quality single-nucleus transcriptomic profiles enabled the identification and annotation of 11 distinct primary cell types, including myelinating SCs (myeSCs), non-myelinating SC (nmSCs), SAMs, monocytes, T cells, natural killer (NK) cells, fibroblasts, endothelial cells (ECs), and plasma cells (Fig. 3a, b). A small subset of SCs and SAMs displayed a high-proliferation phenotype—defined by elevated expression of the proliferation-associated genes *MKI67*, *NUSAP1*, and *TOP2A*—across four VS subtypes, indicating the presence of a functionally conserved proliferative reservoir shared by both cell populations.

We observed significant differences in cell composition across the four VS subtypes. IDSs exhibited a markedly elevated proportion of nmSCs compared with IESs (Fig. 3c). By contrast, IESs showed a significantly increased proportion of myeSCs, accompanied by greater infiltration of immune cells, consistent with prior studies [17–19]. Deconvolution analysis of bulk RNA-sequencing data further confirmed the enrichment of nmSCs in IDSs and the pronounced accumulation of SAMs in IESs (Fig. 3d; Supplementary Fig. 3c, d). *NF2*-SWN IESs exhibited the highest proportion of myeSCs among the four VS subtypes. These findings indicate the cellular heterogeneity across four VS subtypes.

Genomic characterisation of VSs revealed a high frequency of chromosome 22q (chr22q) deletions and *NF2* alterations, along with a low mutational burden [28]. Thus, we performed inferCNV analysis to assess the loss of chr22q at single-cell resolution. IDSs exhibited increased chr22q loss deletion frequencies in SC populations compared with IESs (Fig. 3e, f), suggesting that divergent genomic architectures underlie the molecular subgroups of VSs. Furthermore, chr22q loss was detected in most SAMs and monocytes within *NF2*-SWN IDSs, which may reflect their relatively low abundance in this subtype. Additionally, approximately 75% of fibroblasts harboured a chr22q loss in both *NF2*-SWN IESs and IDSs. This finding supports the hypothesis that tumour-associated fibroblasts in *NF2*-SWN VSs may derive from endoneurial fibroblasts, which share a common neural crest progenitor with SCs during peripheral nerve development [29].

CellChat analysis revealed significantly enhanced intercellular communication in IESs relative to IDSs (Fig. 3g). Ligand-receptor interactions analysis showed that CSF1–CSF1R signaling from SCs to monocytes/SAMs as preferentially enriched in IESs (Supplementary Fig. 3e), potentially driving macrophage recruitment and proliferation. Conversely, IDSs were characterized by dominant SPP1–integrin (ITGA4/ITGB1) interactions between SCs and monocytes, which may support adhesion-mediated survival (Supplementary Fig. 3e). In addition, MHC-II, GAS, BMP, and IL-16 signaling pathways were predominantly enriched in IESs, while VEGF, WNT, and SLITRK pathways characterized IDS communication networks (Fig. 3h). These divergent signaling profiles underscore subgroup-specific therapeutic vulnerabilities targeting either immune-stromal crosstalk in IESs or SC-autonomous proliferation in IDSs.

### Dynamics of SC trajectories recapitulate the stages of nerve injury and repair

**Fig. 4:**
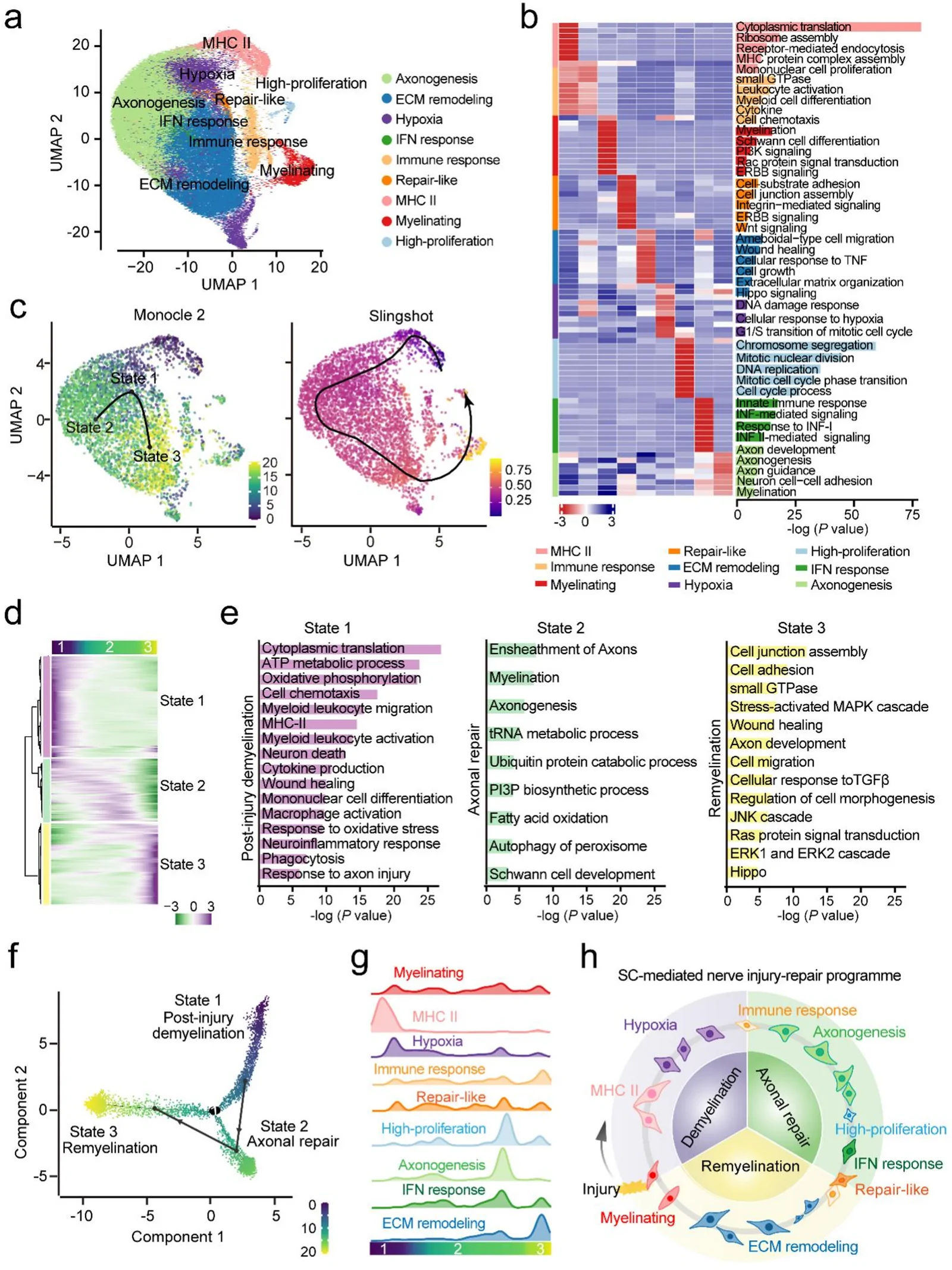
SC subclusters display heterogeneous transcriptional profiles. **a** UMAP projection of SCs revealing nine subclusters in VSs. **b** Heatmap of subcluster-specific marker genes (left) and enriched pathways (right). Colour intensity indicates relative expression; bar length represents −log_10_(*P* value). **c** UMAP projections of Monocle 2 (left) and Slingshot (right) pseudotime trajectories. Black curves, fitted trajectory paths; arrows, directionality. **d** Trajectory-based partitioning of SCs into three functional states. **e** Gene ontology enrichment analysis for each pseudotime state: State 1 (post-injury demyelination), State 2 (axonal repair), and State 3 (remyelination). **f** Monocle 2 pseudotime trajectory elucidating dynamic transitions among SC subpopulations. **g** Ridge plots depicting cell density distribution of SC subclusters across pseudotime. **h** Schematic illustration of the SC-mediated nerve injury and repair programme

To investigate the functional diversity of SCs in VSs, 70,066 SCs were computationally resolved into nine distinct subclusters based on their gene signatures (Fig. 4a; Supplementary Fig. 4a). In addition to myeSCs (e.g., *PRX*,* MPZ*), nmSC subclusters included high-proliferation (e.g., *MKI67*), axonogenesis (e.g., *SCN7A*,* SLC24A2*), extracellular matrix (ECM) remodelling (e.g., *FOSB*,* PTX3*), hypoxia (e.g., *VEGFA*), IFN response (e.g., *IFI44L*,* MX2*), repair-like (e.g., *RUNX2*), and major histocompatibility complex class II (MHC-II) (e.g., *HLA-DRA1*) SCs. Notably, axonogenesis SCs and ECM remodelling SCs (38.7% and 32.4%, respectively) constituted the two largest subpopulations in VSs (Supplementary Fig. 4b), suggesting that SCs predominantly activate transcriptional programmes characteristic of peripheral nerve injury and repair.

To systematically integrate our findings with previously published scRNA-seq datasets and enable cross-study comparability, we performed bidirectional label transfer and functional re-annotation using two independent reference datasets [17, 18]. This approach ensured consistent SC nomenclature across studies (Fig. 4b; Supplementary Fig. 4c, d). For example, we renamed the “core” and “stress” subsets described previously as axonogenesis and ECM remodeling subclusters, respectively—thereby more precisely reflecting their distinct functional roles in axon development and extracellular matrix organization.

To assess whether SCs mirror the temporal dynamics of nerve injury and regeneration, we reconstructed SC differentiation trajectories using two independent algorithms (Monocle 2 and Slingshot). Monocle 2 analysis showed two distinct linear paths and one bifurcation event separating divergent functional lineages, suggesting the presence of three transcriptionally and functionally distinct cellular states (Fig. 4c). Slingshot analysis independently validated this trajectory architecture, identifying a dominant lineage trajectory extending from State 1 to State 3 with high pseudotime coherence. The DEGs were clustered along the pseudotemporal axis, and the signalling pathway dynamics associated with each SC state were mapped (Fig. 4d, e). We found that State 1 enriched for energy metabolism, myeloid cell recruitment, macrophage activation, and axon injury response; State 2 dominated by ensheathment of axons, axonogenesis, and SC development; and State 3 was characterised by cell adhesion, migration, and axon development. Strikingly, these states mirrored the canonical temporal stages of peripheral nerve regeneration following injury (Fig. 4f), including post-injury demyelination, axonal repair, and remyelination stages [30, 31]. These findings further confirm that SCs retain the core transcriptional programmes underlying physiological nerve injury and repair in VSs.

To identify key regulators governing SC state transitions, we analysed gene expression dynamics along the pseudotemporal trajectory (Supplementary Fig. 4e). We found sustained high expression of *JUN* across three stages, consistent with its role in driving the transition from myeSCs to nmSCs following nerve injury [32]. By contrast, *SOX2*, a negative regulator of myelination [33], showed progressive downregulation in the axonal repair and remyelination stages. *NRG1* expression increased continuously, peaking during the remyelination state, whereas *IL34* displayed transient upregulation during the axonal repair stage followed by downregulation. These complementary expression patterns collectively support a model in which SCs undergo a temporally ordered progression—from an initial injury-responsive state to terminal differentiation.

Notably, we also observed distinct temporal enrichment patterns of SC subclusters across the pseudotemporal states (Fig. 4g). The post-injury demyelination stage was predominantly characterised by MHC-II and hypoxia SCs, whereas axonogenesis SCs marked the axonal repair stage, and ECM remodelling SCs defined the remyelination stage. These findings demonstrate that SC subclusters within the VS microenvironment undergo precise transcriptional reprogramming to orchestrate the spatiotemporal stages of peripheral nerve injury and repair (Fig. 4h).

### Divergent SC trajectories across the four VS subtypes

**Fig. 5:**
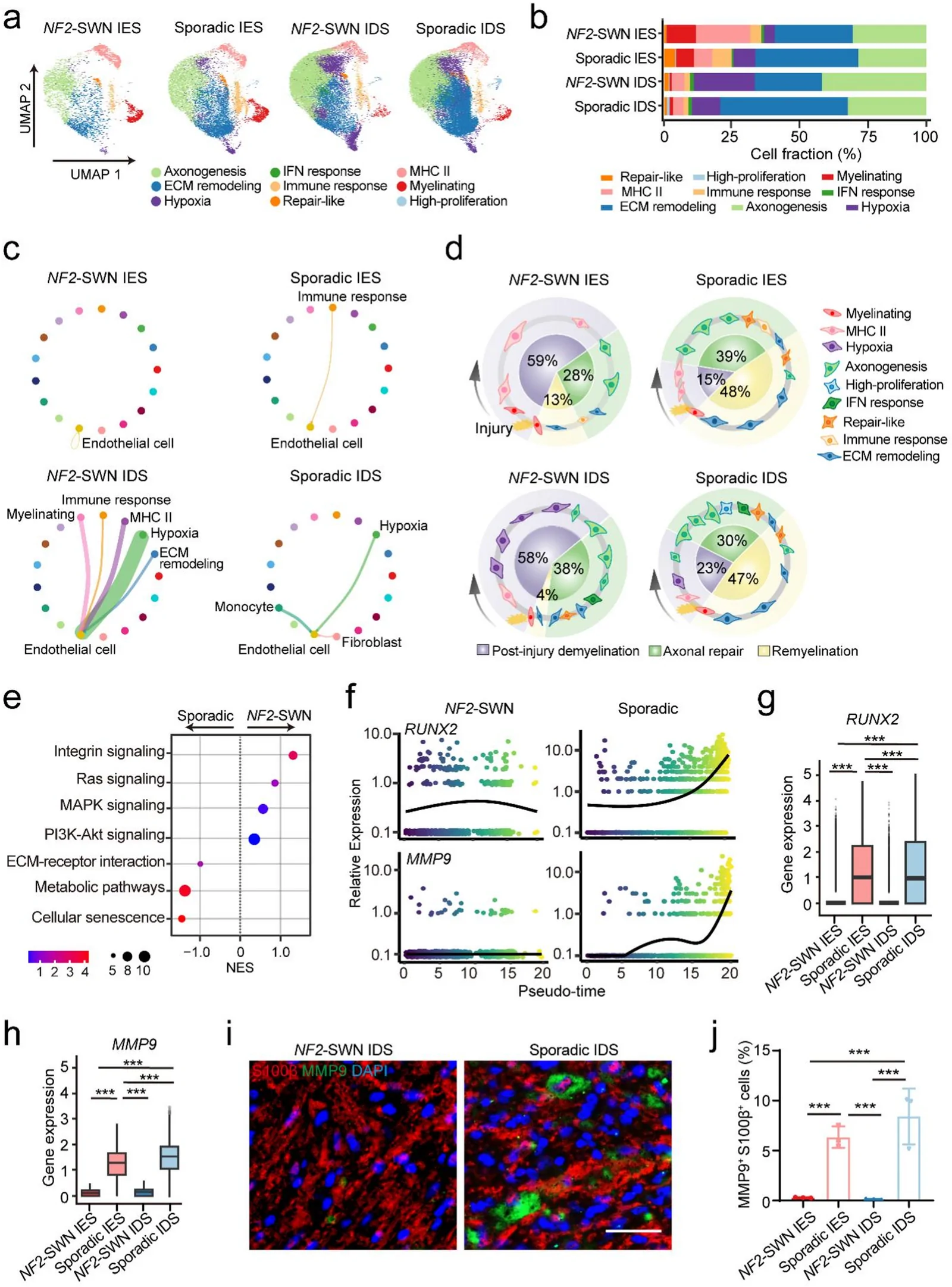
Schwann cell injury–repair dynamics differ between *NF2*-SWN and sporadic VSs. **a** UMAP projection illustrating SC functional states across four molecular subtypes. **b** Stacked bar plots showing proportions of functional states across four molecular subtypes. **c** Circle plots showing VEGF signaling interactions across four molecular subtypes. Circle size, cell number; edge width, communication probability. **d **Circular diagram depicting the injury–repair cycle in each subtype: the inner ring represents the three repair states, and the outer ring shows nine functional programmes. Percentages reflect the distribution of cells across states. **e** Gene Set Enrichment Analysis (GSEA) comparing *NF2*-SWN and sporadic VSs. Dot colour corresponds to the normalised enrichment score (NES), and dot size indicates gene set size. **f** Kinetic curves of *RUNX2* and *MMP9* expressions along pseudotime in SCs. **g** Box plot of *RUNX2* expression across four molecular subtypes. Statistical significance: ***P < 0.001 (two-way ANOVA). **h** Box plot of *MMP9* expression across four molecular subtypes. Statistical significance: ***P < 0.001 (two-way ANOVA). **i** Representative immunofluorescence images showing co-staining for MMP9 (green) and S100β (red) in *NF2*-SWN and sporadic IDSs, with DAPI nuclear counterstain (blue). Scale bars, 20 μm. **j** Quantification of MMP9^+^ S100β^+^ cells as a percentage of total cells (*n* = 3 per group). Data are mean ± SD. Statistical significance: ***P < 0.001 (one-way ANOVA)

We compared the SC composition across the four VS subtypes and found significantly higher proportions of myeSCs in *NF2*-SWN and sporadic IESs (11.2% and 7.0%, respectively) compared with IDSs (0.4% and 1.3%, respectively) (Fig. 5a, b; Supplementary Fig. 5a). *NF2*-SWN IESs had the highest proportion of MHC-II SCs (21.0%), whereas hypoxia SCs were predominant in *NF2*-SWN IDSs (23.7%). Sporadic IESs exhibited the highest frequency of repair-like SCs (7.7%), whereas sporadic IDSs displayed the largest proportion of ECM remodelling SCs. Moreover, *NF2*-SWN IESs exhibited a lowest frequency of chr22q loss in SC subclusters among four VS subtypes (Supplementary Fig. 5b). MHC-II SCs showed a significantly lower proportion of chr22q loss than all other SC subclusters across all VS subtypes. This finding suggests that preservation of the wild-type NF2 allele or relative genomic stability is essential for maintaining their immunomodulatory capacity of this subcluster.

CellChat analysis uncovered distinct intercellular communication patterns across SC subclusters among the four molecular subtypes of VSs. *NF2*-SWN IDSs exhibited enhanced VEGF signaling-mediated crosstalk between hypoxia SCs and endothelial cells (Fig. 5c). In contrast, sporadic IESs displayed a unique CNTN1–NOTCH1 interaction network involving multiple SC subclusters and SAMs (Supplementary Fig. 5c). Trajectory analysis revealed distinct pseudotemporal progression patterns of SCs between *NF2*-SWN and sporadic VSs. In *NF2*-SWN VSs, SCs exhibited profound developmental arrest within the postinjury demyelination stage, characterised by enrichment of MHC-II SCs in IESs and hypoxia SCs in IDSs (Fig. 5d; Supplementary Fig. 5d). By contrast, sporadic VSs showed robust trajectory progression toward the remyelination stage, where ECM remodelling SCs predominated in both sporadic IESs and IDSs. These divergent trajectories reflect fundamentally distinct pathophysiological states in *NF2*-SWN SCs versus sporadic SCs.

To further delineate the functional heterogeneity of SCs, we compared the single-cell transcriptome profiles of SCs from *NF2*-SWN VSs versus sporadic VSs. Functional enrichment analysis of DEGs revealed upregulation of signalling pathways associated with integrin, Ras, and MAPK signalling pathways in *NF2*-SWN VSs, indicating constitutive activation of pro-migratory and pro-survival signalling cascades in *NF2*-SWN SCs (Fig. 5e). By contrast, sporadic VSs displayed the activation of signalling pathways related to ECM-receptor interaction, metabolism, and cellular senescence. To further investigated the mechanisms underlying ECM remodelling in VSs, we screened the DEGs enriched in ECM-receptor interaction pathway. We identified significant higher level of *MMP9* and its transcription factor *RUNX2* in sporadic VSs compared with *NF2*-SWN VSs (Fig. 5f–h). We then performed IF staining and confirmed significant higher MMP9 protein expression in sporadic VSs than in *NF2*-SWN VSs (Fig. 5i, j). Given that both runt-related transcription factor 2 (RUNX2) and MMP9 are regulators of SC differentiation and migration [34–36], our data support a critical role of RUNX2–MMP9 axis in SC-mediated remyelination and highlight its therapeutic potential in VS treatment.

### Divergent functional states of monocytic lineage cells across the four VS subtypes

**Fig. 6:**
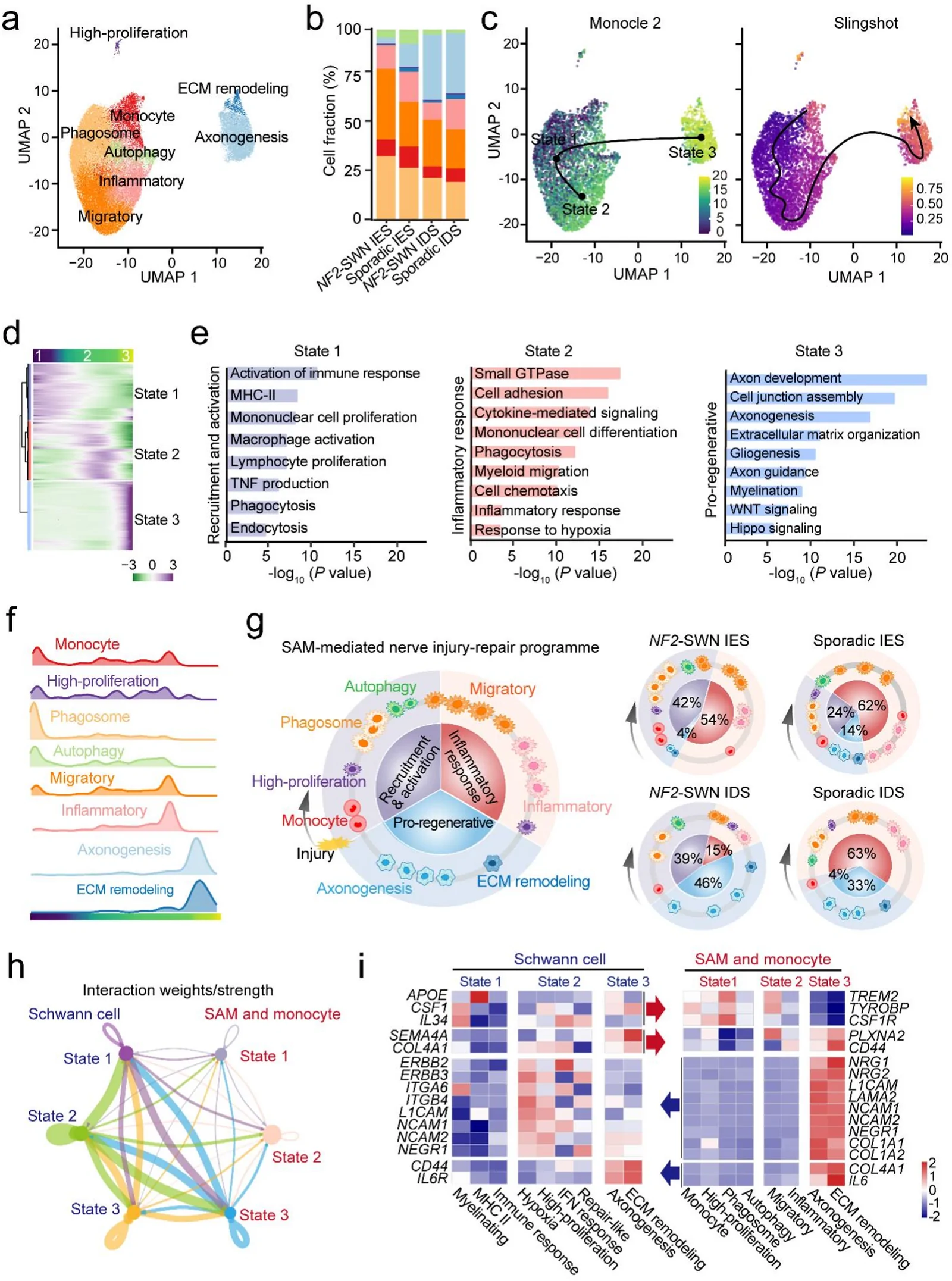
Distinct distribution of SAMs and monocytes across four VS subtypes. **a** UMAP projection of MCs revealing eight subclusters (phagosome, migratory, inflammatory, autophagy, axonogenesis, ECM remodelling, monocyte, proliferation) in VSs. **b** Stacked bar plots showing proportions of myeloid cell subclusters across four VS subtypes. **c** UMAP projections of Monocle 2 (left) and Slingshot (right) pseudotime trajectories in SAMs and monocytes. Black curves fitted trajectory paths; arrows, directionality. **d **Trajectory-based partitioning into three states. **e** Gene ontology enrichment for each state: State 1 (recruitment and activation), State 2 (inflammatory response), and State 3 (pro-regenerative). **f** Ridge plots depicting myeloid cell subcluster density across pseudotime. **g** Schematic representation of the SAM and monocyte-mediated cycle for nerve injury-repair model. Right, **c**ircular diagrams depicting cycle progression and state distribution percentages in each subtype. **h** Interaction network among SC states (States 1–3) and SAM and monocyte states (States 1–3). Node size corresponds to the proportion of each cell state; edge width and colour intensity reflect interaction strength. Bidirectional arrows indicate reciprocal signalling interactions. **i** Heatmap illustrating ligand–receptor pair expression between SC and SAM and monocyte states. Left: ligands specific to the SC state (rows) are shown across SC functional programmes (columns), with red arrows indicating outgoing signalling toward SAM and monocyte states. Right: ligands specific to the SAM and monocyte state (rows) are displayed across SAM and monocyte functional programmes (columns), with blue arrows indicating incoming signalling originating from SC states. Colour scale represents row-normalised expression (Z-score)

To investigate monocytic lineage cells heterogeneity in VSs, we analysed snRNA-based gene expression profiling of monocytic lineage cells (*n* = 49,821) and identified nine distinct subclusters, including monocytes and eight subsets of SAMs (Fig. 6a; Supplementary Fig. 6a, b). We annotated SAM subclusters as phagosome, migratory, inflammatory, autophagy, axonogenesis, high-proliferation, and ECM remodelling SAMs based on their specific biological processes [18, 37] and harmonized this annotation scheme with our previous study (Supplementary Fig. 6c) [17]. We observed phagosome and migratory SAMs were the two predominant subclusters in both *NF2*-SWN and sporadic IESs, whereas axonogenesis SAMs accounted for more than 30% of the SAMs in IDSs (Fig. 6b), suggesting a fundamental divergence in myeloid cell functional states between two subgroups.

We also reconstructed pseudotime trajectories of myeloid cells to delineate the developmental relationships among these functionally distinct subclusters. Monocle 2 and Slingshot analyses identified three sequential transcriptional states: State 1 (recruitment and activation stage), State 2 (inflammatory response stage), and State 3 (pro-regenerative stage) (Fig. 6c–e). These states mirror the sequential stages of peripheral nerve injury and repair, with proinflammatory mediators (*CD86*,* CTSB*,* VEGFA*,* HIF1α*) peaking during the inflammatory response stage and pro-regenerative genes (*NCAM1*,* NRG1*) restricted to the repair stage (Supplementary Fig. 6d).

State-specific mapping revealed distinct compartmentalization: monocytes, phagosome and autophagy SAMs predominated in State 1; migratory and inflammatory SAMs characterized State 2; and axonogenesis and ECM remodelling SAMs enriched State 3 (Fig. 6f). Notably, trajectory analysis revealed strikingly divergent state distributions of monocytic lineage cells across four VS subtypes. IESs were characterised by predominant State 2 accumulation, with *NF2*-SWN IESs additionally maintaining substantial State 1 populations (42%) (Fig. 6g; Supplementary Fig. 6e). In contrast, myeloid cells in IDSs exhibited pronounced enrichment in State 3 (> 30%). Intriguingly, sporadic IDSs displayed a distinctive bifurcated distribution of monocytic lineage cells across States 2 and 3, accompanied by marked underrepresentation in State 1 (4%). This suggests that monocytic lineage cells in sporadic IDSs have shifted from the initial recruitment phase toward a self-sustaining chronic inflammatory milieu that coexists with active nerve repair programmes.

Systematic ligand–receptor interaction analysis revealed robust bidirectional crosstalk between SCs and SAMs. SCs across all pseudotemporal states exhibited predominant interaction with SAMs and monocytes in State 3, mediated by nerve repair-associated signalling axes, including SEMA4A–PLXNA2 and COL4A1–CD44 (Fig. 6h, i). Conversely, axonogenesis and ECM remodelling SAMs served as the dominant signalling source to SCs, engaging them through well-established nerve repair pathways, including NRG–ERBB, NCAM1/2, and IL6–IL6R [38, 39]. These findings indicate that pro-regenerative SAMs establish a trophic niche that actively supports SC proliferation and differentiation, thereby sustaining nerve repair programmes within the VS microenvironment.

### Divergent lymphocyte fates within TME across four VS subtypes

**Fig. 7:**
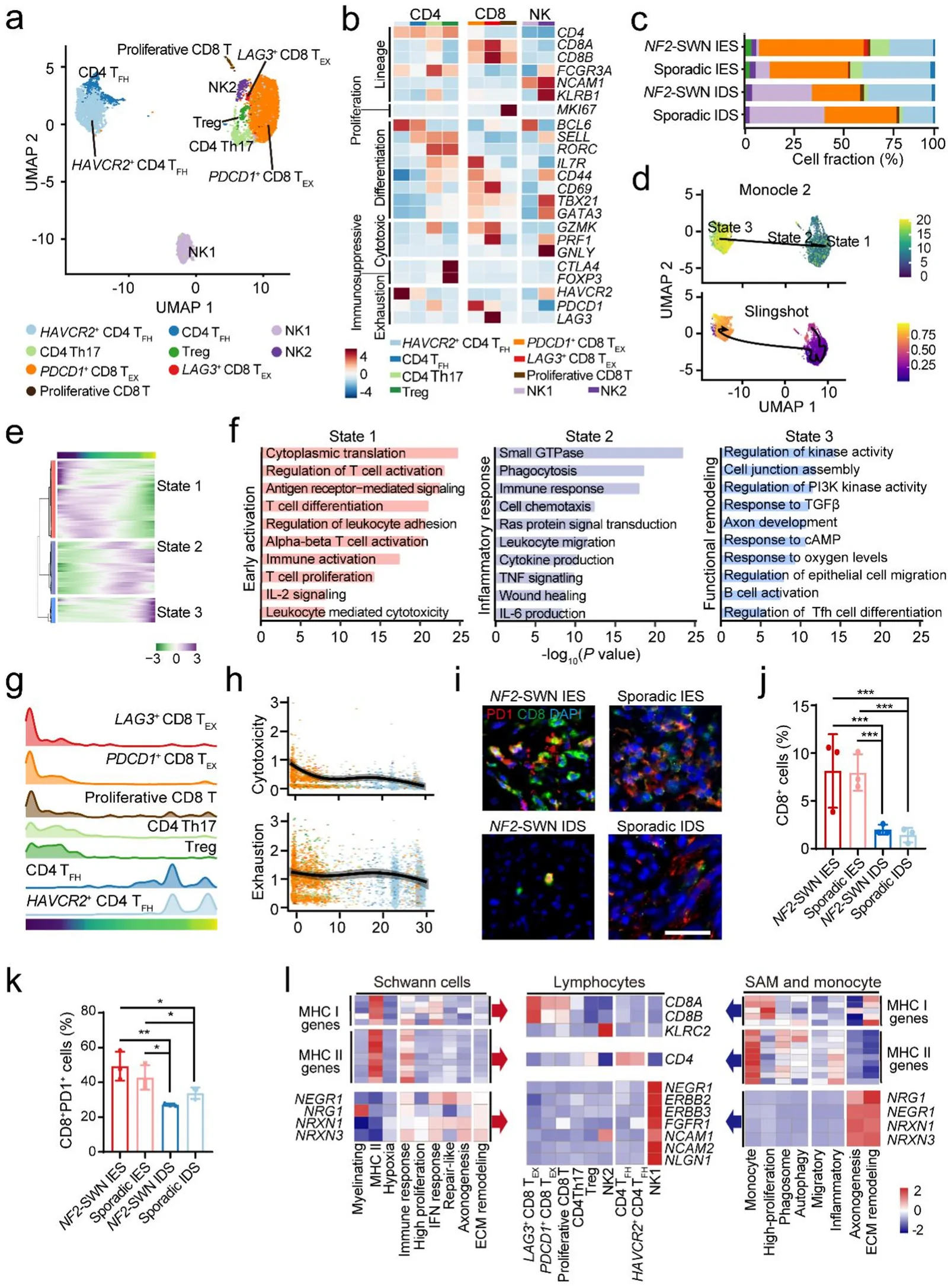
Distinct T cell subclusters exhibit heterogeneous transcriptional profiles. **a** UMAP projection of T cells revealing nine subclusters in VSs. **b** Heatmap displaying subcluster-specific marker genes across T cell lineages (CD4^+^ T cell, CD8^+^ T cell and natural killer [NK] cell), including markers for lineage identity, proliferation, differentiation, cytotoxicity, immunosuppression, and exhaustion. **c** Stacked bar plots showing proportions of functional states across four VS subtypes. **d** UMAP projections of Monocle 2 (top) and Slingshot (bottom) pseudotime trajectories in T cells. Black curves, fitted trajectory paths; arrows, directionality. Color scale, pseudotime value. **e** Trajectory-based partitioning into three states. **f** Gene ontology enrichment showing top signalling pathways for each state: State 1 (activation and effector), State 2 (follicular expansion), and State 3 (lymphoid neogenesis). **g** Ridge plots showing the density distribution of T cell subclusters along pseudotime. **h** Pseudotemporal dynamics of cytotoxicity and exhaustion scores across T cell trajectories. **i** Representative immunofluorescence images of co-staining of CD8 (green) and PD-1 (red) in VS samples. Nuclei were counterstained with DAPI (blue). Scale bars, 20 μm. **j** Quantification of CD8^+^ T cells as a percentage of total cells (*n* = 3 per group). Data are mean ± SD. Statistical significance: ***P < 0.001 (one-way ANOVA). **k** Quantification of CD8^+^PD-1^+^ T cells as a percentage of total CD8^+^ T cells (*n* = 3 per group). Statistical significance: *P < 0.05, **P < 0.01 by one-way ANOVA. **l** Heatmap of ligand–receptor pair expression profiles. Left: ligands associated with SC States across functional programmes (red arrows indicate outgoing signalling to T cells). Middle: ligands associated with T-cell states. Right: ligands linked to MC states across functional programmes (blue arrows represent incoming signalling from SCs). Colour scale: row-normalised Z-score

Subclustering analysis of 6,406 T and NK cells using reference-based classification with canonical molecular markers and cell type-specific transcriptional signatures [40–42] revealed nine distinct subclusters: four CD4^+^ T cell subsets (T helper 17 cells [T_H_17], T follicular helper cells [T_FH_], *HAVCR2*^+^ T_FH_ cells, and regulatory T cells [Tregs]), three CD8^+^ T cell subsets (exhausted *PDCD1*^+^ T cells [T_EX_], *LAG3*^+^ T_EX_ cells, and proliferative T cells), and two NK cell subsets (NK1 and NK2) (Fig. 7a, b; Supplementary Fig. 7a, b). Interestingly, NK1 cells displayed a unique transcriptional signature enriched for axon regeneration-related genes, implicating a specialized role in peripheral nerve injury and repair.

Across the four VS subtypes, *NF2*-SWN IESs harboured the highest proportion of *PDCD1*^+^ CD8^+^ T_EX_ cells (55.4%) and the lowest proportion of NK1 cells (1.3%), whereas sporadic IESs exhibited the highest frequency of *HAVCR2*^+^ CD4 T_FH_ cells (55.4%) **(**Fig. 7c; Supplementary Fig. 7c, d). By contrast, NK1 cells constituted more than 30% of the lymphocytes in both *NF2*-SWN and sporadic IDSs. These findings indicate that the VS microenvironment orchestrates functionally distinct immunological niches across molecular subtypes.

Reconstruction of T cell differentiation trajectories revealed a sequential bifurcated differentiation programme. State 1 was defined by activated effector T cells, whereas States 2 and 3 comprised T_FH_ cells and Tregs exhibiting chronic inflammatory response and functional remodeling (Fig. 7d–g). Moreover, most T cells across all VS subtypes predominantly exhibited an activation and effector signature with terminal exhaustion (Fig. 7b, h), suggesting that persistent antigen exposure drives T cell dysfunction and impairs effective antitumour immunity in the VS microenvironment. Consistently, IF staining identified PD-1^+^CD8^+^ T cell infiltration in all VS subtypes (Fig. 7i). Notably, the frequency of PD-1^+^CD8^+^ T cells was significantly higher in IESs than in IDSs (Fig. 7j, k), suggesting the enhanced immunosuppressive milieu in IESs.

To dissect the cellular crosstalk underlying T cell exhaustion, we performed systematic ligand–receptor interaction analysis. This revealed that MHC class I- and MHC class II-mediated signals predominantly originated from MHC-II SCs, monocytes, and high-proliferation SAMs (Fig. 7l), suggesting that persistent antigen presentation contributes to T cell dysfunction. Notably, NK1 cells received robust intercellular signals from the axonogenesis and ECM remodelling SAMs through the NRG1–ERBB, NCAM1–NRXN1, and NRXN–NCAM axes. These findings suggest that SCs and myeloid cells serve as immuno-trophic intermediaries within the VS microenvironment, thereby orchestrating divergent lymphocyte fates.

### Development of an IHC-based classifier for molecular subgrouping

**Fig. 8:**
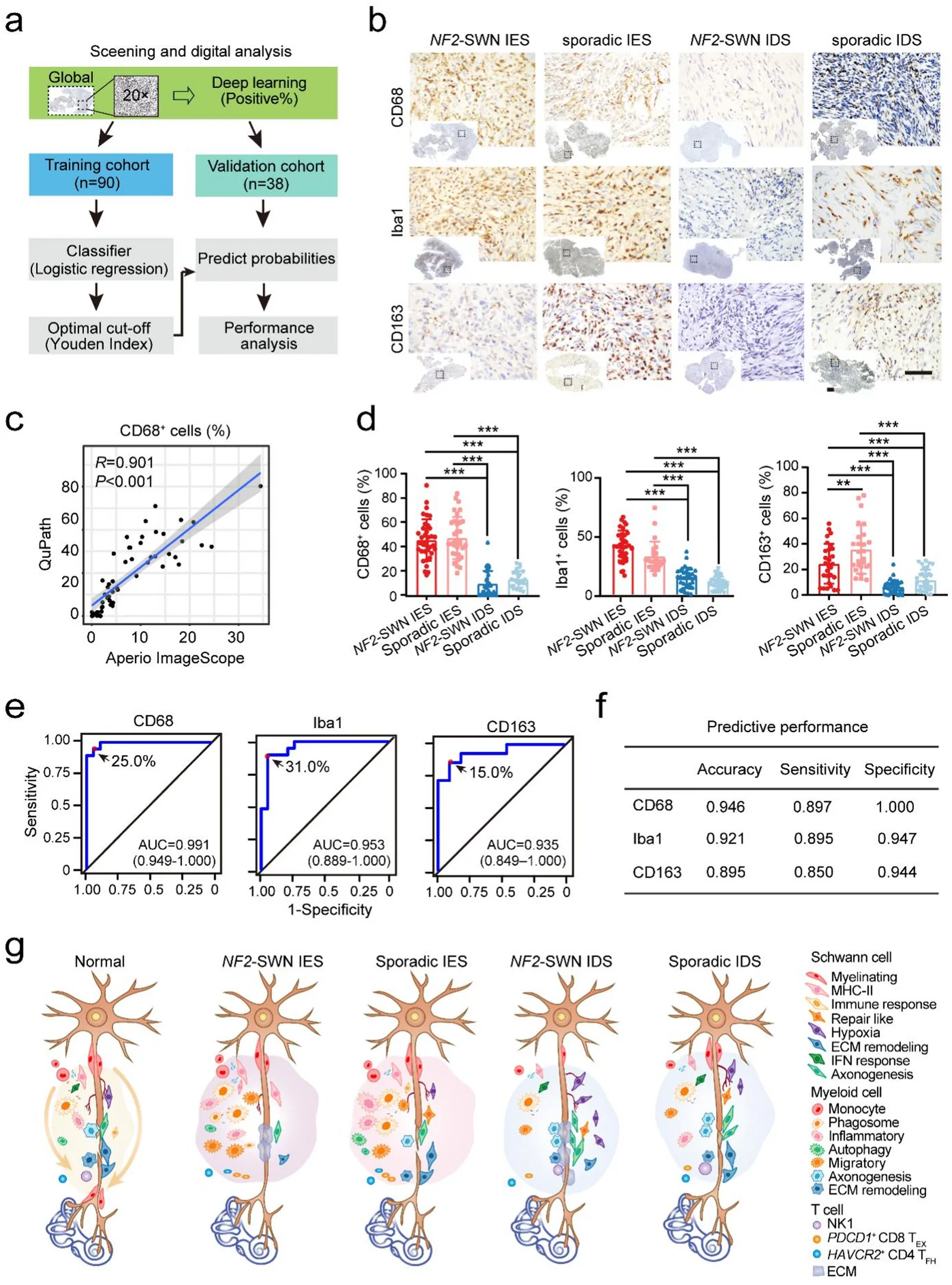
IHC staining revealing the heterogeneity of macrophage infiltration in VSs. **a** Study design and flow diagram. **b** Representative images of IHC staining for antibodies against Iba1, CD68, and CD163 across four VS subtypes. Scale bars, overview = 1 mm; detail = 50 μm. **c** Correlation analysis of CD68⁺ cell percentages between the QuPath and Aperio ImageScope quantification platforms (Spearman correlation, *R* = 0.901, *P* < 0.001). **d** Bar plots showing the percentage of positive cells for CD68, Iba1, and CD163 antibodies across four VS subtypes. Data are mean ± SD. Statistical significance: **P < 0.01, ***P < 0.001 (one-way ANOVA). **e** Receiver operating characteristic (ROC) curves for CD68, Iba1, and CD163 in the validation cohort, with the AUC and 95% confidence intervals indicated. Optimal cut-off points (Youden index) are marked (red spot). **f **Table of performance metrics for CD68, Iba1, and CD163 markers. **g** Schematic illustration of tumour microenvironment heterogeneity across four VS subtypes

Given the differences in SAM infiltration between the two molecular subgroups of VSs, we sought to evaluate whether IHC staining using macrophage-specific antibodies could reliably distinguish these subgroups. IHC staining for Iba1, CD68, and CD163 antibodies was performed on 78 VSs (Fig. 8a, b), supplemented by previously published IHC data from 50 *NF2*-SWN VSs [17]. To quantify the total number of positively stained cells in each slide, we developed a deep learning model using QuPath software (v0.6.0). To assess the consistency of deep learning-based interpretation across imaging platforms, we compared Iba1-, CD68-, and CD163-positive cell detection and quantification results generated using QuPath with those obtained using the Aperio ScanScope XT platform. High concordance was observed between the two platforms, with coefficients of determination of 0.901, 0.827, and 0.768 for Iba1, CD68, and CD163 staining, respectively (Fig. 8c; Supplementary Fig. 8a). We compared the proportions of Iba1-, CD68-, and CD163-positive cells across the four VS subtypes and observed higher proportions of all three macrophage markers in IESs compared with IDSs (all *P* < 0.001; Fig. 8d).

We randomly assigned 128 samples to training (*n* = 90) and validation (*n* = 38) cohorts and applied logistic regression to develop a predictive model for molecular classification using the training cohort. ROC curve analysis demonstrated that Iba1, CD68, and CD163 staining exhibited excellent predictive performance in distinguishing the two subgroups in both the training cohort and the validation cohort (AUCs: 0.991, 0.953 and 0.935, respectively) (Fig. 8e, f; Supplementary Fig. 8b). These results suggest that IHC staining of macrophage biomarkers may serve as a reliable molecular surrogate for identifying the classification of VSs.

## Discussion

The molecular and cellular mechanisms underlying VS initiation and progression remain poorly understood [10]. Hence, clinically validated biomarkers for predicting tumour growth and guiding individualised therapeutic selection are lacking. In this study, we demonstrated that two robust molecular subgroups of VSs exhibit fundamentally distinct transcriptomic and DNA methylation profiles, irrespective of syndromic status. We developed multigene and IHC-based classifiers compatible with routine clinical trial settings. Single-cell analysis uncovered dynamic phenotypic transitions in SCs, macrophages, and T cells that functionally mirror the sequential stages of nerve injury and repair within the VS TME (Fig. 8g). These aberrant injury and repair programmes emerge as critical determinants of VS pathogenesis, driving intertumoural heterogeneity and tumourigenic progression. Our findings establish a unifying paradigm for understanding schwannoma tumourigenesis and the cellular mechanisms of peripheral nerve regeneration.

Our study demonstrates that TME heterogeneity was strongly associated with divergent clinical outcomes across the four VS subtypes. Our findings suggest that the sustained inflammatory milieu in IESs confers pliable biophysical properties that facilitate GTR and their favourable nerve function preservation. By contrast, SC and immune cell-driven ECM remodelling in IDSs results in dense collagenous fibrosis and intimate adhesion to vestibulocochlear or facial nerve fascicles, features that impede complete resection and increase the risk of neurological morbidity and recurrence. These findings confirm molecular classification as a robust preoperative biomarker for predicting surgical resectability and functional neurological outcomes. Therefore, future studies should prioritise the development of MRI-based predictive models to inform individualised treatment strategies, including observation, surgery, or appropriate medical therapy. Additionally, although targeting ECM sensors and remodellers has shown limited efficacy in cancers [43, 44], modulating this axis may represent a promising strategy to improve hearing function or enhance resectability in patients with IDSs.

This study provides novel evidence for the “failure-of-nerve regeneration” theory of schwannoma development. SCs are the predominant glial cell type in the PNS and are essential for peripheral nerve repair and regeneration. After injury, SCs exhibit remarkable cellular plasticity, enabling their functional transition to a “repair phenotype” and orchestrating the progression of the nerve repair programme [45, 46]. Conversely, SC dysfunction, such as loss of Merlin and chr22q deletion, compromises this regenerative capacity and is causally implicated in schwannoma pathogenesis [47, 48]. In this study, we found that the majority of SCs in VSs adopt phenotypes associated with defined stages of the nerve injury and repair, rather than uncontrolled proliferation. Concurrently, immune cells in the VS microenvironment are similarly organised around injury and repair transcriptional programmes and display a dynamic coordinated activation with specific SC subpopulations. These findings support the model wherein VSs represent chronic unhealed wounds of the vestibular nerve, characterised by a sustained inflammatory milieu shaped by interactions between SCs and immune cells. Therefore, future studies should further uncover the mechanisms governing aberrant SC plasticity and reprogramming and identify instructive signals from the surrounding inflammatory microenvironment that can be targeted to control tumour growth.

Our data reveal that VSs are intrinsically heterogeneous tumours harbouring distinct cellular ecosystems. To date, the mechanisms underlying this intertumoural heterogeneity remain incompletely characterised, as existing single-cell transcriptomic studies have been restricted to limited cell numbers and confined to sporadic tumours [18–20, 49, 50]. We found higher proportions of myeSCs and MHC-II SCs in IESs, accompanied by a lower incidence of chr22q loss, compared with IDSs. Notably, both SC subclusters contribute to the recruitment of immune cells and sustain their proliferation within the VS microenvironment. This suggests that preservation of the wild-type *NF2* allele in immunomodulatory SC subpopulations serves as the foundational mechanism, representing a key molecular determinant underlying the divergent cellular ecosystems observed in IESs versus IDSs. Notably, the APOE–TREM2/TYROBP signalling axis was found to be a key mediator of intercellular crosstalk between MHC-II SCs and proinflammatory SAMs, establishing it as a novel therapeutic target for suppressing macrophage-mediated inflammation in IESs.

Our results also demonstrate that the developmental trajectories of SCs diverged significantly between *NF2*-SWN and sporadic VSs. The majority of SCs in *NF2*-SWN VSs exhibited a “locked” injury-response state relative to those in sporadic VSs. This persistent state likely arises from the polyclonal growth and multilobulated architecture of *NF2*-SWN VSs [51, 52], in which asynchronous expansion of spatially distinct clonal foci along the vestibular nerve may generate chronic, multifocal injury signals, thereby arresting SCs in the post-injury demyelination stage. Moreover, the chronic, widespread tumour burden across both the peripheral and central nervous systems in patients with *NF2*-SWN [53] contributes to a systemic inflammatory milieu that may promote a “chronically unhealed wound” phenotype. Additionally, the absence of imperative signals from *NF2*-deficient neurons likely impairs SC remyelination in patients with *NF2*-SWN [48]. By contrast, sporadic VSs, which typically present in late adulthood, are characterised by SCs that progress to the remyelination stage; however, elevated cellular senescence in SCs may compromise axonal regeneration [54, 55]. Overall, these novel insights highlight the distinct molecular mechanisms underlying SC behaviour across the four VS subtypes and underscore the importance of considering SC heterogeneity in developing targeted therapeutic strategies.

We further revealed dynamic differentiation programmes of SAMs that converge to the “chronically unhealed wound” phenotype of VSs. These programmes initiate with macrophage recruitment and activation, followed by a transition to a sustained proinflammatory effector state. These findings indicate that SAMs function as the primary cellular effectors of myelin and axonal debris clearance and then undergo substantial proliferation and recruitment in the VS microenvironment, particularly in IESs [17, 48, 56]. By contrast, IDSs exhibited significant enrichment of two SAM subclusters characterised by pro-regenerative phenotypes. These subclusters play a critical role in orchestrating nerve regeneration, including modulating the activity of SCs and NK1 cells through the secretion of trophic factors. Given that the underlying mechanisms of human macrophages in nerve regeneration remain incompletely assessed [48], our study establishes a robust framework for deciphering the molecular crosstalk between macrophages, SCs, and T cells—a foundation that could enable selective modulation of this tripartite interaction to either suppress tumour growth or promote macrophage-mediated nerve regeneration.

Moreover, lymphocytes are central effectors of antitumour immunity and contribute to immune responses associated with tissue repair, whereas the role of lymphocytes in peripheral nerve regeneration remains unclear [57, 58]. A recent study of *NF2*-SWN VSs demonstrated both CD4^+^ and CD8^+^ T cells displayed terminal differentiation phenotypes [59]. In line with their findings, our data also showed that majority of effector T cells undergo progressive functional exhaustion following infiltration into the VS microenvironment. Notably, our data provide novel evidence for T_FH_ cell-driven formation of lymphoid-like structures in VSs, particularly in sporadic IESs, suggesting a potential association between ectopic lymphoid neogenesis and tumour growth [60]. Thus, investigating the functional roles of distinct T cell subsets within VSs may enhance our understanding of cellular immune responses across various stages of peripheral nerve repair and inform the development of immunomodulatory therapeutic strategies for VSs.

This study had several limitations. First, due to the limited sample size of our snRNA-seq dataset, the observed TME composition may not be broadly generalizable. Second, the absence of neutrophils in our snRNA-seq dataset likely reflects the technical sensitivity limits of snRNA profiling for low-RNA-content cells [61, 62]. This limitation underscores the urgent need for complementary spatial or immunohistochemical approaches to fully resolve the neutrophil landscape and its spatial heterogeneity in VSs [63].

In conclusion, our study delineates the dynamic cellular heterogeneity across the four molecular subtypes of VSs, elucidating distinct molecular and cellular mechanisms that underlie tumour development and progression. These findings identify modulation of SC–immune cell crosstalk as a promising therapeutic avenue with the dual potential for tumour control and peripheral nerve regeneration.

## Supplementary Information

Supplementary Material 1.

Supplementary Material 2.

Supplementary Material 3.

## Data Availability

The bulk RNA-sequencing and snRNA-seq data generated in this study have been deposited at GSA-Human (https://ngdc.cncb.ac.cn/gsa-human/) under accession GSA-Human (https://ngdc.cncb.ac.cn/gsa-human/browse/HRA002762; https://ngdc.cncb.ac.cn/gsa-human/browse/HRA016927). The raw counts from snRNAseq and bulk RNAseq along with raw methylation data have been released on Zenodo (https://doi.org/10.5281/zenodo.21324866). Analytical code is publicly accessible via GitHub (https://github.com/BNI-code/multi-omic-analysis.git). All data supporting the findings in this study are available from the lead contact (F.Z.) upon reasonable request.

## Abbreviations

VS: Vestibular schwannoma
*NF2*-SWN: *NF2*-related schwannomatosis
CPA: Cerebellopontine angle
VEGF: Vascular endothelial growth factor
SC: Schwann cell
PNS: Peripheral nervous system
snRNA-seq: Single-nucleus RNA sequencing
TME: Tumour microenvironment
IES: “Immune-enriched” schwannoma
IDS: “Immune-depleted” schwannoma
SAMs: Schwannoma-associated macrophages
MRI: Magnetic resonance imaging
FFPE: Formalin-fixed paraffin-embedded
AAO-HNS: American Academy of Otolaryngology–Head and Neck Surgery
cDNA: Complementary DNA
PCR: Polymerase chain reaction
t-SNE: t-distributed stochastic neighbour embedding
NMF: Non-negative matrix factorisation
DEGs: Differentially expressed genes
KEGG: Kyoto Encyclopedia of Genes and Genomes
DMPs: Differentially methylated positions
FDR: False discovery rate
GSEA: Gene set enrichment analysis
NES: Normalised enrichment score
AUC-ROC: Area under the receiver operating characteristic curves
IHC: Immunohistochemistry
Iba1: Ionised calcium-binding adaptor molecule-1
OD: Optical density
IF: Immunofluorescence
MMP9: Matrix metalloproteinase 9
ROI: Region of interest
CI: Confidence interval
SD: Standard deviation
UHC: Unsupervised hierarchical clustering
myeSCs: Myelinating SCs
nmSCs: Non-myelinating SCs
NK: Natural killer
ECs: Endothelial cells
ECM: Extracellular matrix
MHC-II: Major histocompatibility complex class II
RUNX2: Runt-related transcription factor 2
IQR: Interquartile range
ROC: Receiver operating characteristic
UMAP: Uniform manifold approximation and projection
DOS: Duration of symptoms
GTR: Gloss total removal
Pre.: Preoperative
Post.: Postoperative
STR: Subtotal resection
yrs: Years
