# Supplementary material for: Divergent Schwann cell–immune cell trajectories across vestibular schwannoma subtypes mirror nerve injury-repair programs
Source: J Neuroinflammation. 2026 Aug 3;23:360. doi: 10.1186/s12974-026-03995-w (PMC13721222; doi:10.1186/s12974-026-03995-w)
Supplement: Supplementary file 1 [file 12974_2026_3995_MOESM3_ESM.pdf]

# Supplementary Material

**Title:** Divergent Schwann cell–immune cell trajectories across vestibular schwannoma subtypes mirror nerve injury-repair programs

**Journal:** Journal of Neuroinflammation

**Authors:** Han-Guang Zhao, Xiao-Ping Wang, Jing Zhang, Si-Kang Ren, Yu-Chao Liang, Hui Li, Jiang-ming Li, Xiao-bin Zhao, Lei-Ming Wang, Yuan-Xiang Lin, Zhi Li and Fu Zhao

**Corresponding authors:** Fu Zhao (zhaofu@ccmu.edu.cn) and Zhi Li (Zhi Li @ccmu.edu.cn), Beijing Neurosurgical Institute, Capital Medical University, 119 South 4th Ring West Road, Fengtai District, Beijing, 100070, PR China, Tel: +8610 59976775, Fax: +8610 59976775. Yuan-Xiang Lin (lyx99070@163.com), The First Affiliated Hospital of Fujian Medical University, Fuzhou, Fujian province, 350005, PR China.

**This PDF file includes:**

Supplementary Figures. 1 to 8

Table S1

## 1. Supplementary Figures

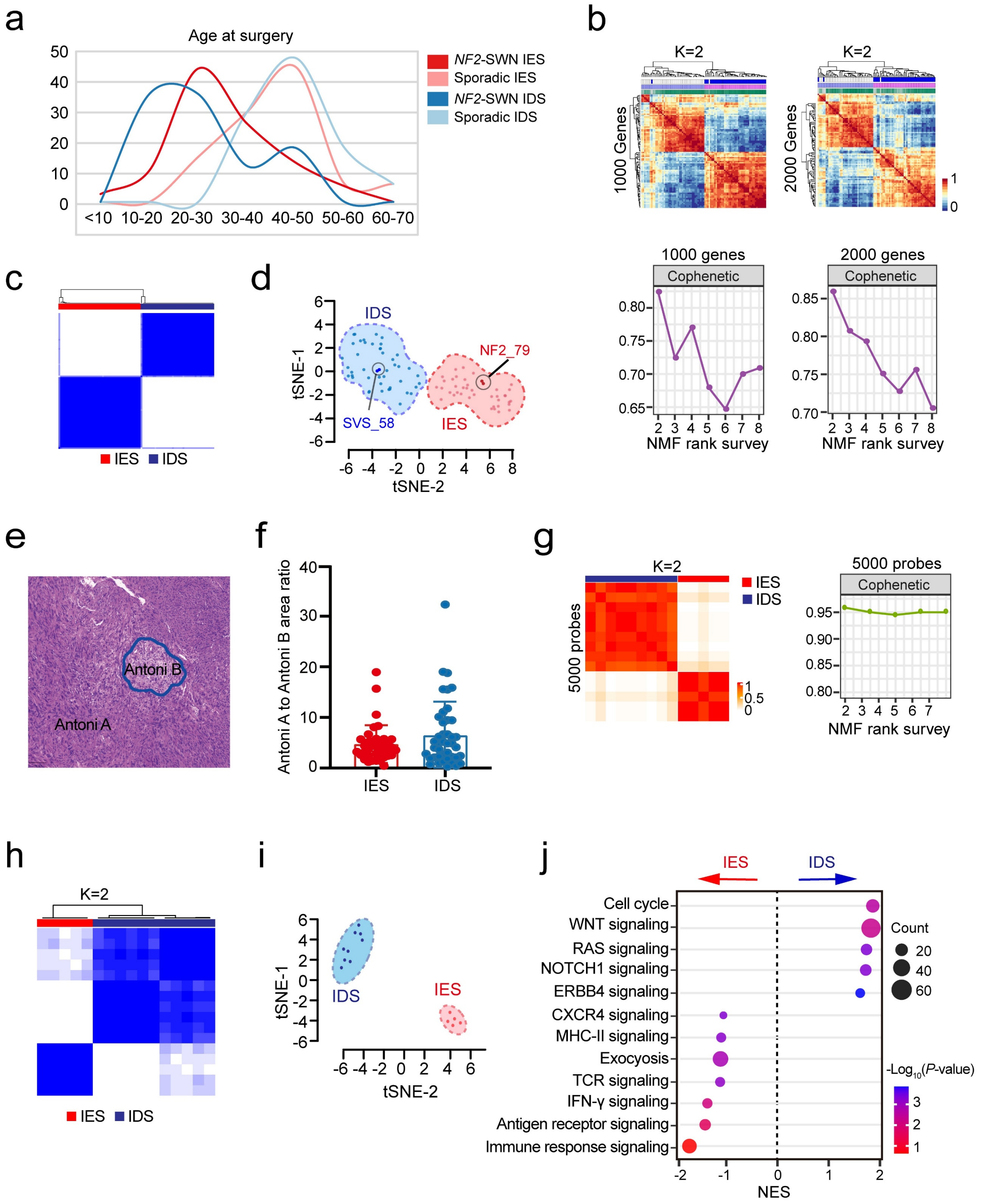


**Fig. S1. Two molecular subgroups in VSs exhibiting distinct DNA methylation profiling. (a)** Distribution of patient age at surgery across four VS subtypes. **(b)** NMF-derived consensus matrices for 1,000 and 2,000 variable transcripts (K = 2). Cophenetic correlation analyses support robust bifurcation

at K = 2. **(c)** Consensus clustering matrix demonstrating two robust molecular subgroups across sporadic and *NF2*-SWN VSs. **(d)** t-Distributed Stochastic Neighbor Embedding (t-SNE) projection with unsupervised cluster assignments. Individual samples are represented as dots; ellipses denote 95% confidence intervals. **(e)** Representative H&E staining showing Antoni A and Antoni B areas; the region of interest (dashed line) indicates Antoni B area quantified for analysis. **(f)** Quantification of Antoni A to Antoni B area ratio in two subgroups**. (g)** NMF consensus heatmap and cophenetic correlation plot for 5,000 most variable CpG probes, indicating robust bifurcation at K = 2. **(h)** Consensus clustering matrix demonstrating two robust methylation-based subgroups across sporadic and *NF2*-SWN schwannomas. **(i)** t-Distributed Stochastic Neighbor Embedding (t-SNE) projection with unsupervised cluster assignments. Individual samples are represented as dots; ellipses denote 95% confidence intervals. **(j)** Gene Set Enrichment Analysis (GSEA) of differentially methylated genes between IES and IDS subgroups using the KEGG database. Bubble size indicates gene count; color scale represents $-\log_{10}$(*P*-value); NES, normalized enrichment score.

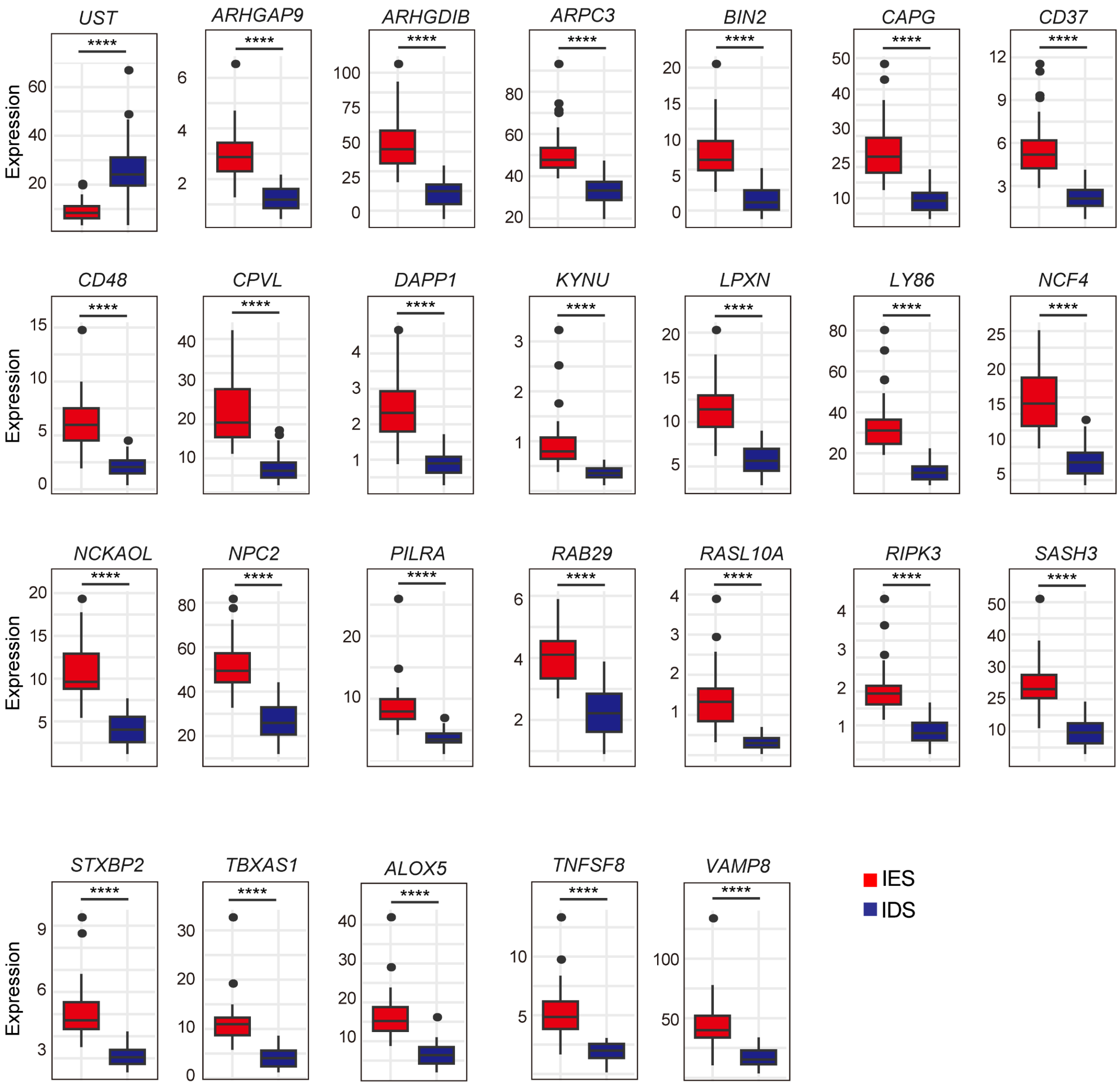


**Fig. S2. The top differentially expressed genes between two molecular subgroups of VSs.** Expression values are log2(TPM+1). Box plots display median (horizontal line) and interquartile range; whiskers extend to 1.5× IQR. Statistical significance: ****$P < 0.0001$ (two-sided Wilcoxon rank-sum test).

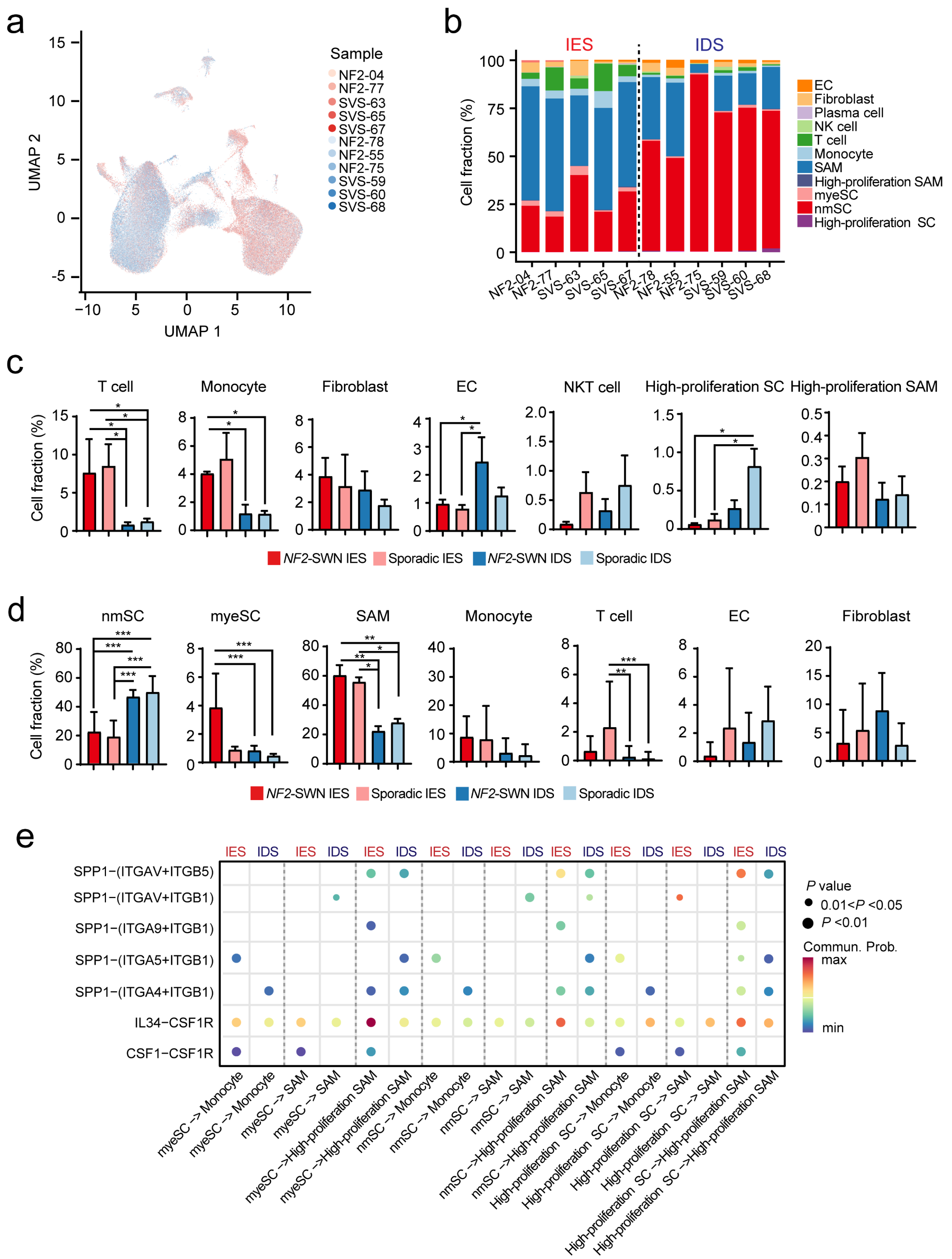


**Fig. S3. snRNA-seq reveals distinct cellular compositions across four molecular subtypes of VSs. (a)** UMAP visualization of 11 transcriptionally distinct cell clusters from 11 VS samples. **(b)** Stacked bar plot

showing cell type proportions across individual samples. Dashed line separates IES and IDS samples. **(c)** Cell type proportions across four molecular subtypes of VSs. Data are presented as mean ± SEM. Statistical significance: $*P < 0.05$, $**P < 0.01$ (two-way ANOVA with Tukey's post-hoc test). **(d)** Quantification of cell type proportions predicted by CIBERSORTx deconvolution of bulk RNA-seq data. Statistical significance: $**P < 0.01$, $***P < 0.001$ by one-way ANOVA. **(e)** CellChat analysis of ligand-receptor interactions between schwann cell subtypes and macrophages. Bubble size, $-\log_{10}(P$ value); colour, interaction strength (communication probability).

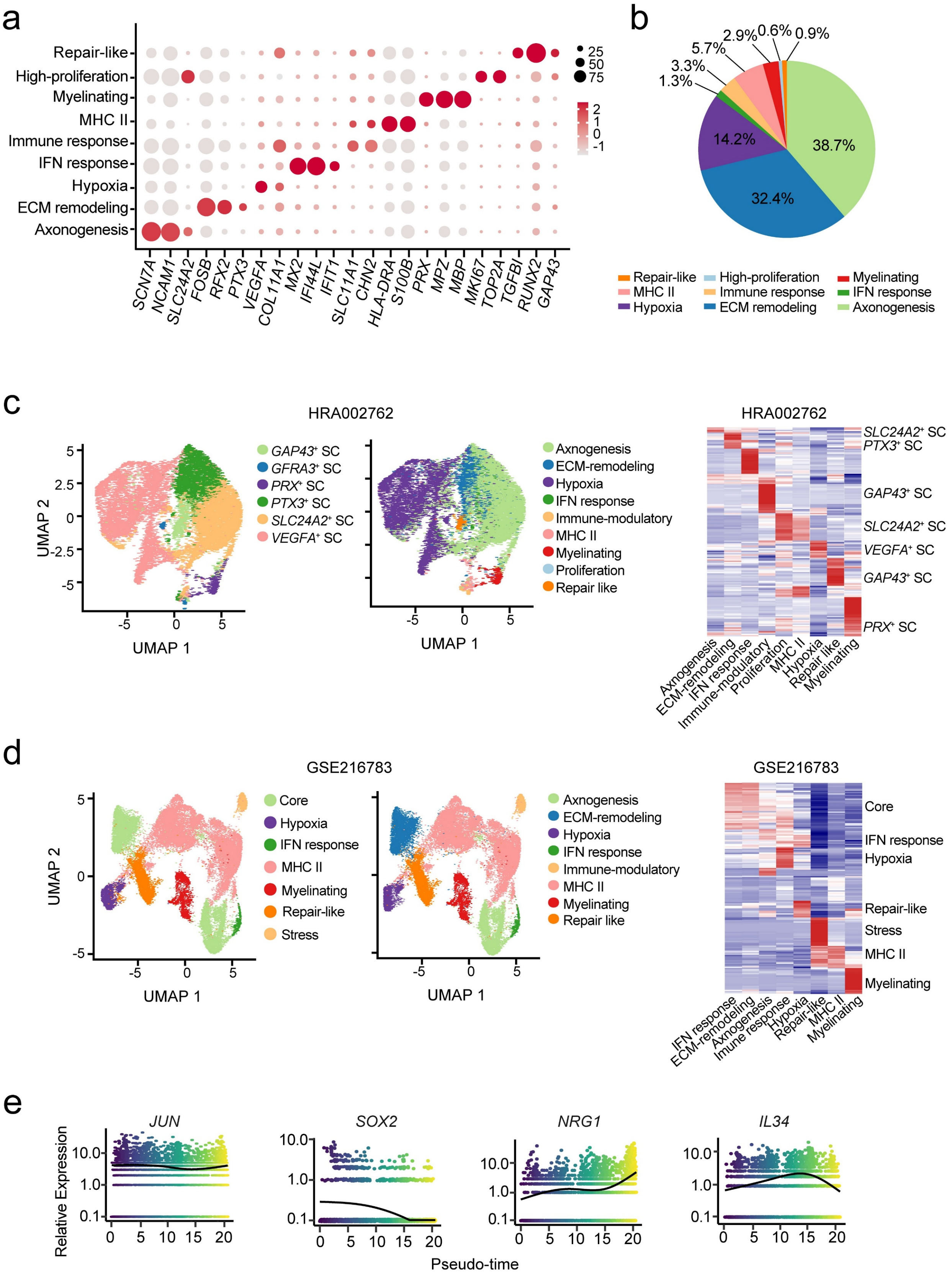


**Figure S4. Distinct SC subclusters display heterogeneous transcriptional profiles. (a)** Dot plot of expression levels of selected marker genes (x-axis) for each SC subcluster (y-axis). Dot size represents the

proportion of cells expressing the gene; color intensity indicates average expression level (scaled). **(b)** Pie chart depicting the proportional distribution of SC subtypes across all analyzed samples. **(c)** UMAP visualization and heatmap of SC subtypes in the HRA002762 dataset. Left, SC subtypes annotated by marker gene expression (*GAP43*$^{+}$, *GFRA3*$^{+}$, *PRX*$^{+}$, *PTX3*$^{+}$, *SLC24A2*$^{+}$, and *VEGFA*$^{+}$ SCs). Middle, functional module assignment after label transfer from HRA016927. Right, scaled expression heatmap of top marker genes across the assigned functional modules (rows, individual genes; columns, functional modules; red, high expression; blue, low expression). **(d)** UMAP visualization and heatmap of SC subtypes in the GSE216783 dataset. Left, original published annotation (Core, Hypoxia, IFN response, MHC II, Myelinating, Repair-like, and Stress). Middle, functional module assignment after label transfer from HRA016927. Right, scaled expression heatmap of top marker genes across functional modules after label transfer and re-annotation (rows, individual genes; columns, functional modules; red, high expression; blue, low expression). **(e)** Kinetic curves of key marker genes along pseudotime: pan-trajectory (*JUN*), demyelination (*SOX2*), remyelination (*NRG1*), and macrophage recruitment (*IL34*).

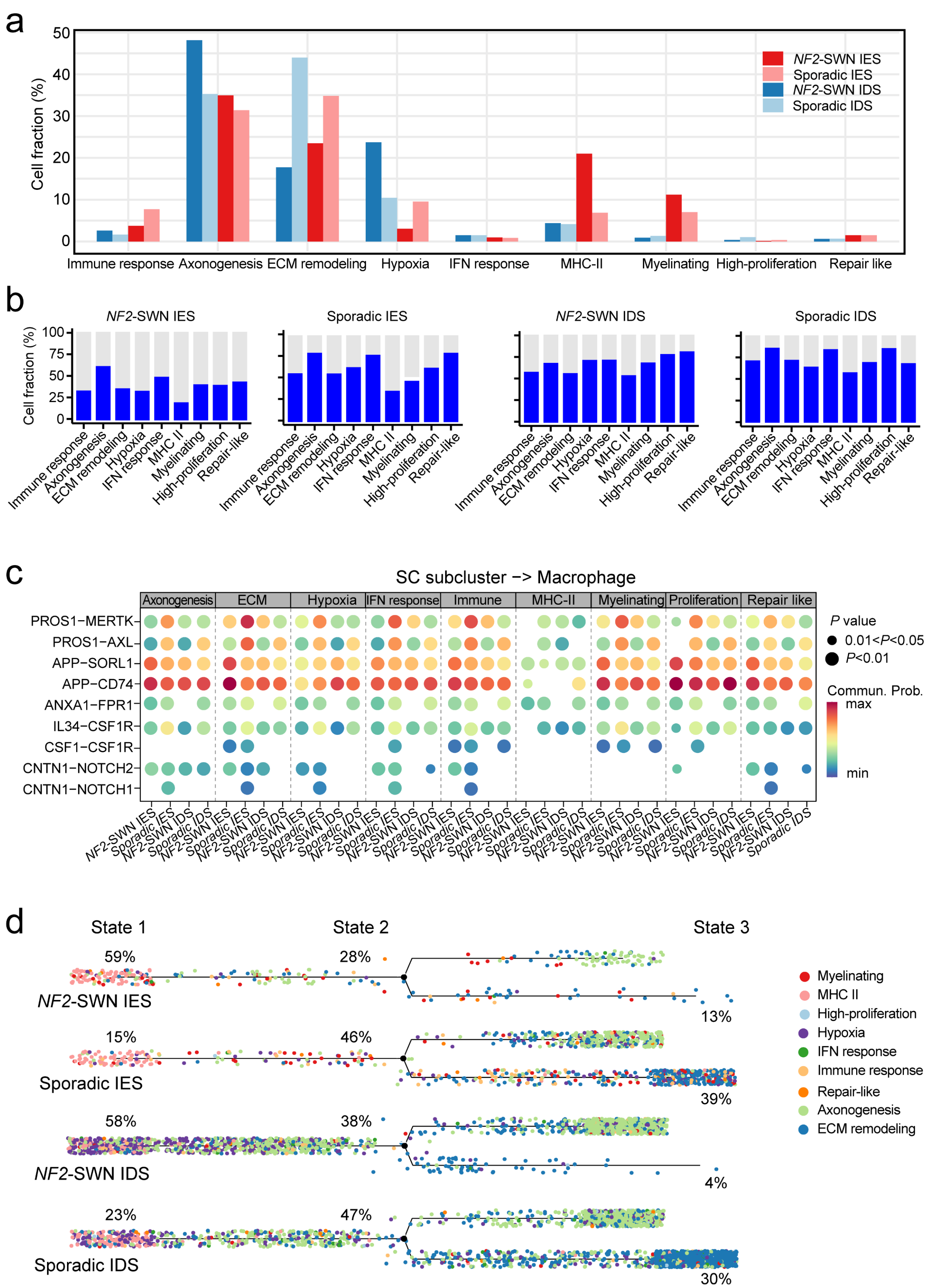


**Figure S5. Schwann cell injury-repair dynamics differ between *NF2*-SWN and sporadic VSs.**

**(a)** Bar plot showing the proportional distribution of nine Schwann cell subtypes across *NF2*-SWN IES, sporadic IES, *NF2*-SWN IDS, and sporadic IDS. **(b) S**tacked bar plot showing the different proportions of chr22q loss in each cell population across different subtypes. **(c)** Cell Chat analysis of ligand-receptor interactions between Schwann cell functional modules and macrophages across molecular subtypes. Bubble size indicates statistical significance $-\log_{10}$ (*P* value); color intensity represents interaction strength (scaled communication probability). **(d)** Pseudotime trajectory analysis illustrates the cellular states across molecular subtypes.

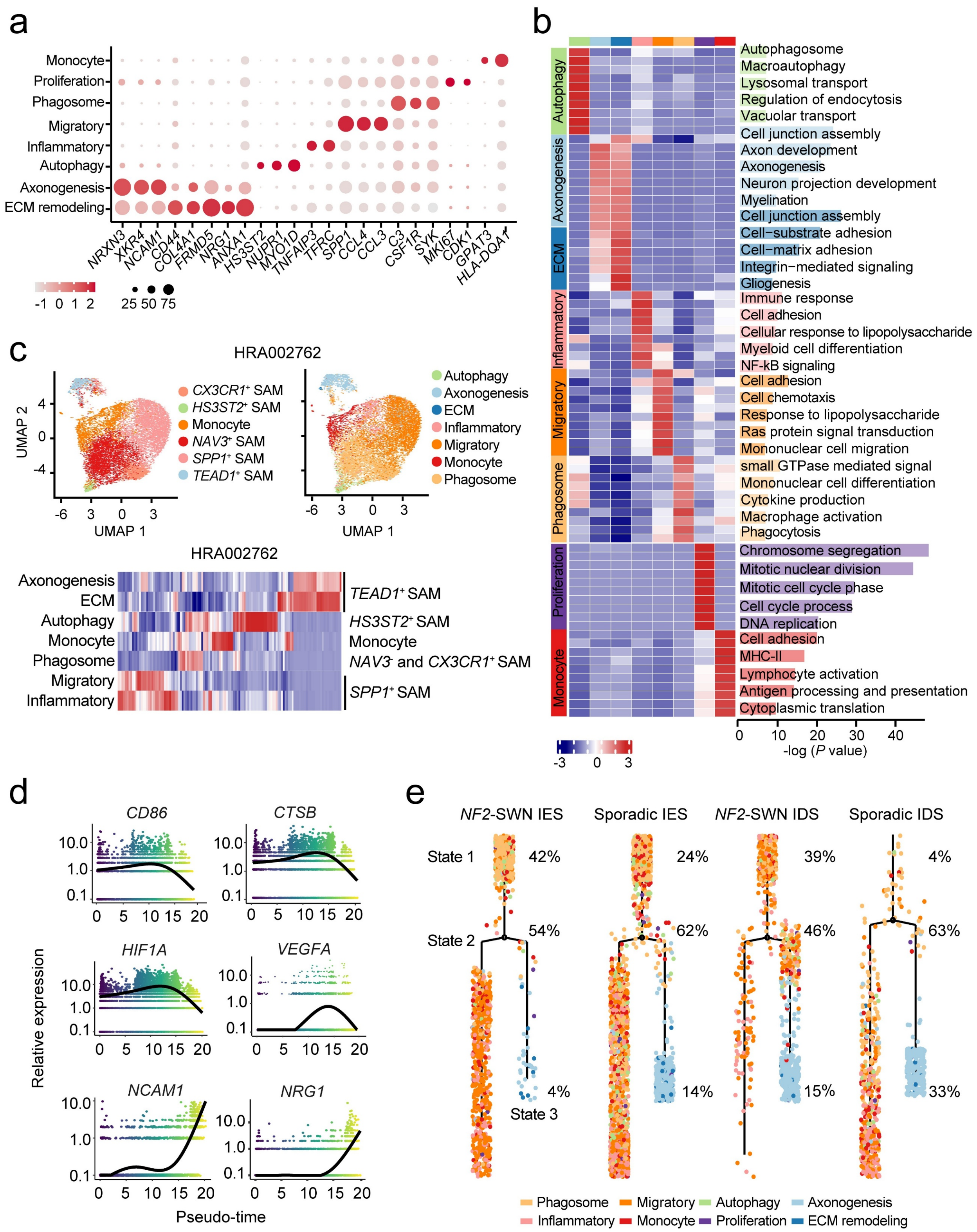


**Figure S6. Heterogeneity of myeloid cell subclusters in VSs. (a)** Dot plot visualization of lineage-specific marker gene expression across myeloid subclusters. The size of each dot indicates the proportion of cells

expressing the gene, while the color intensity represents the average expression level (scaled by z-score). **(b)** Heatmap of subcluster-specific marker genes (left) and Gene Ontology (GO) pathway enrichment analysis (right). Color intensity indicates relative expression; bar length represents $-\log_{10}(P$ value). **(c)** UMAP visualization of SAM subtypes in HRA002762 (left: marker gene-based annotation; right: functional module assignment after label transfer from HRA016927). Heatmap showing scaled expression of top marker genes across functional modules in HRA002762. Rows represent individual genes; columns represent functional modules. Color intensity indicates relative expression level (red: high, blue: low). **(d)** Kinetic curves of key marker genes along pseudotime: inflammatory response (*CD86*, *CTSB*, *HIF1A*, *VEGFA*), axonal repair (*NCAM1*, *NRG1*). **(e)** Pseudotime trajectory analysis illustrates the cellular states across molecular subtypes.

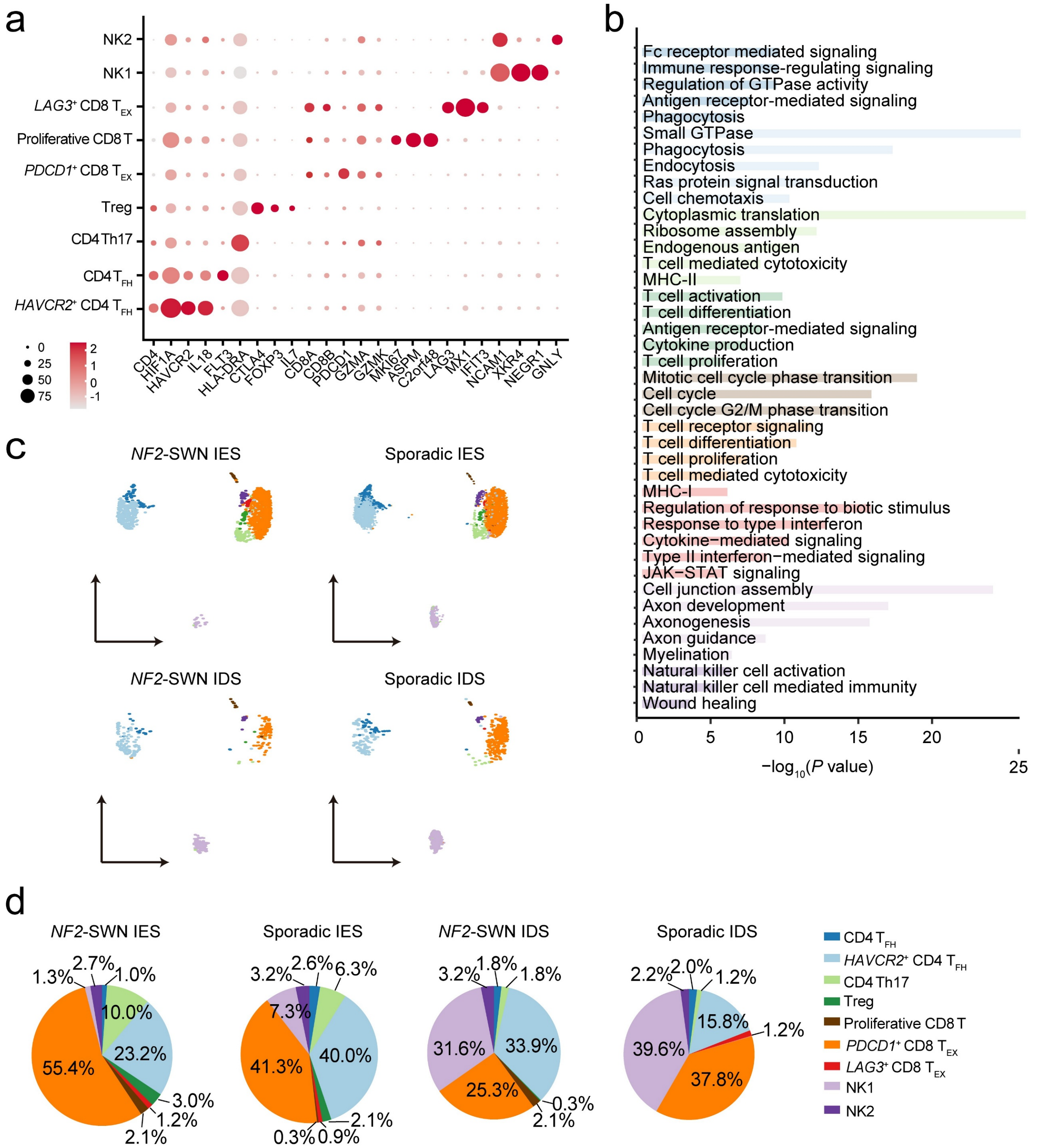


**Figure S7. Distinct T cell subclusters exhibit heterogeneous transcriptional profiles. (a)** Dot plot visualization of lineage-specific marker gene expression across T cell subclusters. The size of each dot indicates the proportion of cells expressing the gene, while the color intensity represents the average expression level (scaled by z-score). **(b)** The bar chart shows Gene Ontology (GO) pathway enrichment analysis (right). Color intensity indicates relative expression; bar length represents $-\log_{10}$(*P* value). **(c)** t-SNE visualization of T cell nine subclusters across four molecular subtypes. **(d)** Pie chart depicting the

proportional distribution of nine T cell subtypes across *NF2*-SWN IES, sporadic IES, *NF2*-SWN IDS, and sporadic IDS.

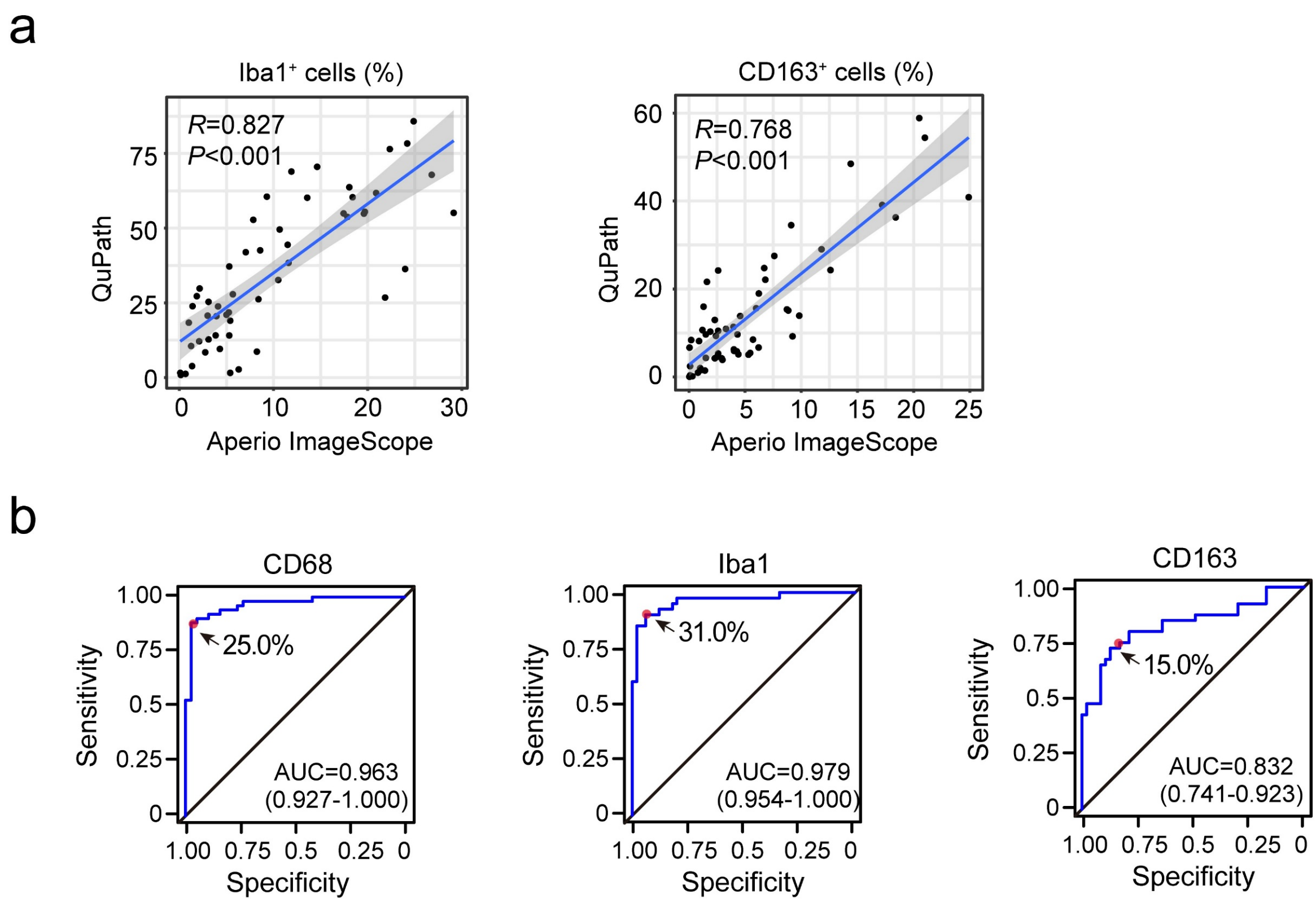


**Figure S8**. **IHC staining reveals the heterogeneity of SAM infiltration in sporadic and *NF2*-SWN VSs.**

**(a)** Inter-platform concordance analysis of Iba1$^+$ and CD163$^+$ cell percentages between QuPath and Aperio ImageScope digital pathology systems (Spearman correlation). **(b)** Receiver operating characteristic (ROC) curves evaluating the diagnostic performance of CD68, Iba1, and CD163 positivity for molecular subtype classification in the training cohort.

**Table S1.** Clinical characteristics of the patient cohort in this study

| Characteristic | All (n = 84) |
|---|---|
| Age, mean (range, year) | 47.2 (11–71) |
| Sex, female | 44 (51.8%) |
| *NF2*-SWN, yes | 18 (21.2%) |
| Tumor size, mean (range, mm) | 33.0 (12–63) |
| Extent of resection, GTR | 79 (92.9%) |
| Lived | 85 (100%) |
| Recurrence | 2 (2.4%) |

*GTR* gloss total removal.
